# Selective recognition of ATP by multivalent nano-assemblies of bisimidazolium amphiphiles through “turn-on” fluorescence response

**DOI:** 10.3762/bjoc.16.223

**Published:** 2020-11-10

**Authors:** Rakesh Biswas, Surya Ghosh, Shubhra Kanti Bhaumik, Supratim Banerjee

**Affiliations:** 1 Department of Chemical Sciences, Indian Institute of Science Education and Research Kolkata, Mohanpur-741246, Nadia, India

**Keywords:** amphiphile, ATP, excimer, multivalency, self-assembly

## Abstract

Bisimidazolium receptors, tagged with chromophoric pyrene at one end and linked to an *n*-alkyl chain at the other, underwent self-assembly in aqueous media depending on the length of the alkyl segment. The amphiphilic derivatives having *n*-decyl or longer chains, formed nano-assemblies with cyanic–green emission resulting from the stacked pyrene chromophores in the aggregates. The presence of positive surface charges on the multivalent aggregates led to ATP binding which was accompanied by a significant increase in the excimeric emission intensity. This provided a convenient way of monitoring ATP binding in a “turn-on” mode and an efficient detection of ATP was achieved in aqueous buffer and also in buffer containing 150 mM NaCl at physiological pH value. Furthermore, the multivalent aggregates demonstrated a significant selectivity in ATP detection over ADP, AMP and pyrophosphate.

## Introduction

Supramolecular anion sensing is an active area of research which has developed tremendously in the last few decades [1–4]. The importance of anion sensing stems from its importance in various practical applications which range from identifying and detecting environmentally toxic anions to medical diagnostics. In recent years, anion responsive materials [5–6] have also emerged as an interesting class of stimuli-responsive materials. Among the different varieties of anionic species, the selective and sensitive detection of bio-anions is particularly important due to their involvement in various biological functions. One of the challenges in the bio-anion detection is that the sensory probes have to be effective under physiological conditions. The highly polar nature of the aqueous media considerably weakens supramolecular interactions such as H-bonding and electrostatic interactions and as a result, it is in general challenging to design high-affinity synthetic probes for anion sensing under such competitive conditions [7]. One of the strategies that has proven to be quite successful in this regard, not only for bio-anions, rather for a variety of several other analytes also, is multivalency [8–11]. It is a principle ubiquitously used in biology to achieve high affinity binding events with examples ranging from protein–carbohydrate interactions to host–pathogen interactions or cell surface adhesion [12–14]. The high affinity originates from the simultaneous interactions of multiple sites in the receptor with their complementary binding partners in the target analyte [15]. One of the ways to design multivalent systems is to connect the receptors through covalent linkages. Conjugated polymers and conjugated polymer electrolytes are prominent examples of covalently constructed multivalent systems and they have been frequently exploited for the highly sensitive detection of a diverse range of target analytes [16–18]. However, the covalent route often requires time consuming synthetic steps and hence, in recent years, self-assembled multivalency has emerged as a suitable alternative [19]. In this methodology, multivalent arrays are built from comparatively smaller binding sites through self-assembly. The smaller molecular units are easier to synthesize and moreover, the morphology and the surface functionalities of the resultant multivalent structures can be tuned in a modular fashion [20–21]. A number of elegant examples of self-assembled multivalent systems targeting biological analytes such as DNA, heparin, proteins, carbohydrates, etc. have been reported in the literature [22–27].

ATP is an important bio-anion that is the energy currency in cells and is involved in a number of essential biological functions including active transport and cell division [28–29]. For ATP detection, metal complexes using metal ions such as Zn(II), Cu(II) and lanthanide ions have been frequently employed [30–35]. A variety of other systems such as aptamers [36–38], conjugated polymers [39–42], quantum dots [43–44] and sensors based on organic receptors such as imidazolium, ammonium, guanidinium [45–52], etc. have also been developed. Imidazolium based synthetic receptors typically utilize a combination of electrostatic and (C–H)···O^−^ hydrogen bonds involving the acidic proton at the 2-position of the imidazolium moiety to bind to ATP and other phosphate analytes [46]. A number of cyclophanes and tweezers [53–56] have been reported possessing the following common structural features: a) the presence of multiple imidazolium groups to overcome the high hydration enthalpy of phosphates and b) the imidazolium groups are connected to aromatic moieties such as anthracene, pyrene, etc. The variation in the luminescence of the aromatic moieties signals the binding event and furthermore, they provide additional stacking interactions with the nucleobases which contributes to the stability of the complexes. In many cases, these stacking interactions provide binding selectivity among the nucleotides [53–56].

**Figure 1:**
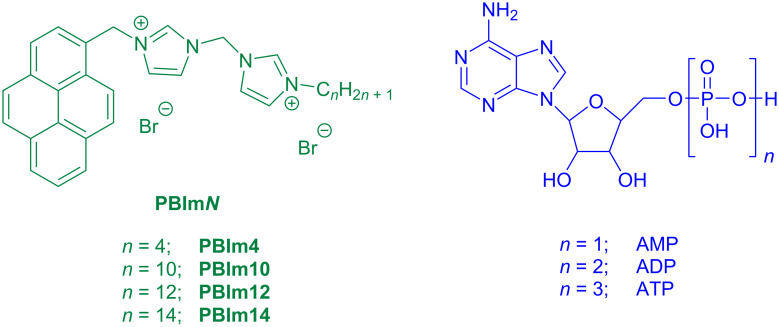
Chemical structures of (a) **PBIm*N*** (*N* = 4, 10, 12 and 14) and (b) ATP, ADP and AMP.

**Scheme 1:**
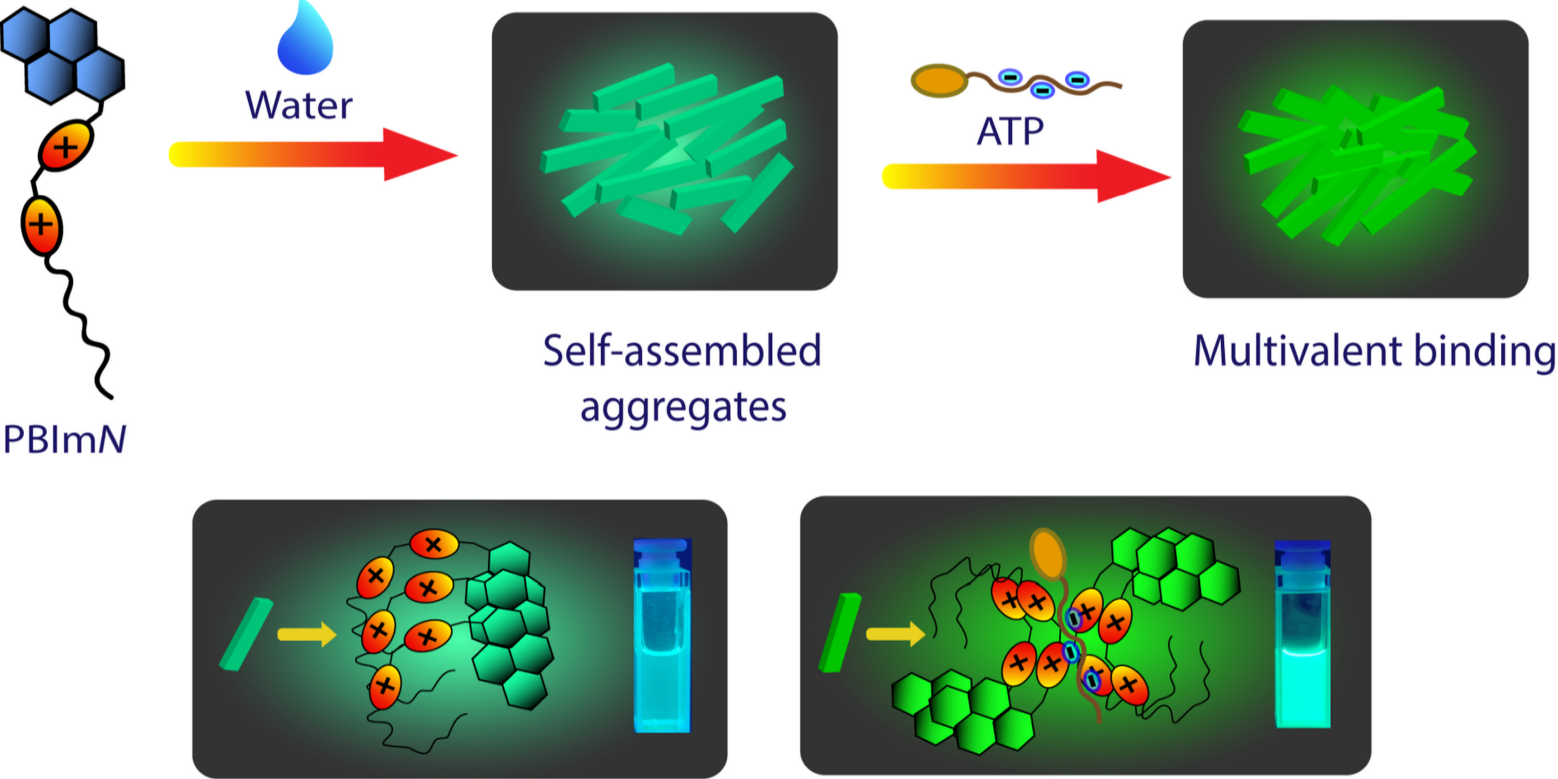
Schematic representation of ATP sensing by multivalent assemblies of **PBIm*N*** in aqueous media.

Most of the aforementioned receptors were predominantly “molecular” in nature with the binding of the nucleotides occurring through multivalent electrostatic interactions in the cavity/cleft created by the spatial orientation of the imidazolium groups. We herein consider a self-assembly based approach in which amphiphilic monomers with bisimidazolium moieties are utilized to create multivalent nano-aggregates for binding to the nucleotides. For this purpose, four pyrene tagged bisimidazolium-based receptors **PBIm*N*** (Figure 1, *N* = 4, 10, 12 and 14; *N* denotes the number of carbons in the *n*-alkyl chain) were synthesized. The longer chain derivatives with *N* ≥ 10 underwent self-assembly in aqueous buffer and produced nanoaggregates with surface exposed positive charges. Interestingly, the aggregates displayed cyanic green luminescence which originated from the excimeric emission of the stacked pyrenes [57–59]. In a recent work, we have demonstrated that self-assembled nanofibrous aggregates of anthracene tagged imidazolium receptors responded to ATP through an amplified fluorescence quenching [60]. Based on these results, we wanted to develop similar self-assembled systems which would show a “turn-on” response in the presence of ATP or other phosphate analytes. The current design of **PBIm*N***s is based on this objective and we found that the excimeric pyrene emissions in these aggregates indeed exhibited a significant increase in the presence of ATP and thus provided a convenient protocol for its detection (Scheme 1). Furthermore, this “turn-on” response was achieved with an appreciable level of selectivity as the response observed in the presence of other adenosine phosphates, ADP and AMP and even pyrophosphate (PPi) was comparatively much lower than that observed for ATP in a wide concentration range (1–100 µM).

## Results and Discussion

### Synthesis

**Scheme 2:**
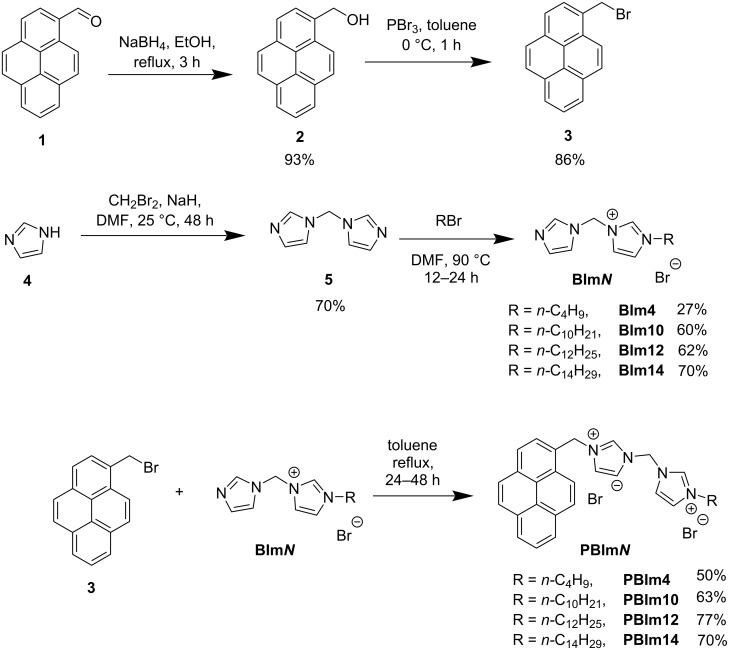
Synthetic route for the preparation of **PBIm*N***s.

The synthesis of **PBIm*N***s (*N* = 4, 10, 12, 14) is depicted in Scheme 2 and the elaborate procedures are given in Supporting Information File 1. The preparation of compounds **2** and **3** are analogous to the procedures from ref. [60]. The pyrene derivative 1-(bromomethyl)pyrene (**3**), which was the common starting material for making all the **PBIm*N***s, was synthesized from pyrene-1-carbaldehyde (**1**) following a literature report [46]. In brief, pyrene-1-carbaldehyde (**1**) was first converted to pyren-1-ylmethanol (**2**) by reduction with sodium borohydride in ethanol [61] and this was followed by treating of **2** with PBr_3_ in toluene to furnish **3**. Di(1*H*-imidazol-1-yl)methane (**5**) was synthesized from imidazole (**4**) and CH_2_Br_2_ following a literature procedure [62–63] with slight modifications. Then, **BIm*N***s (*N* = 4, 10, 12, 14) were synthesized from **5** by mono-alkylation using the corresponding *n*-alkyl halides in DMF. Finally, **PBIm*N***s (*N* = 4, 10, 12, 14) were obtained through nucleophilic substitution reactions between **BIm*N***s (*N* = 4, 10, 12, 14) and **3**. All synthesized compounds were characterized by NMR spectroscopy and high-resolution mass spectrometry.

### Self-assembly properties of PBIm*N*s in aqueous buffer

**Figure 2:**
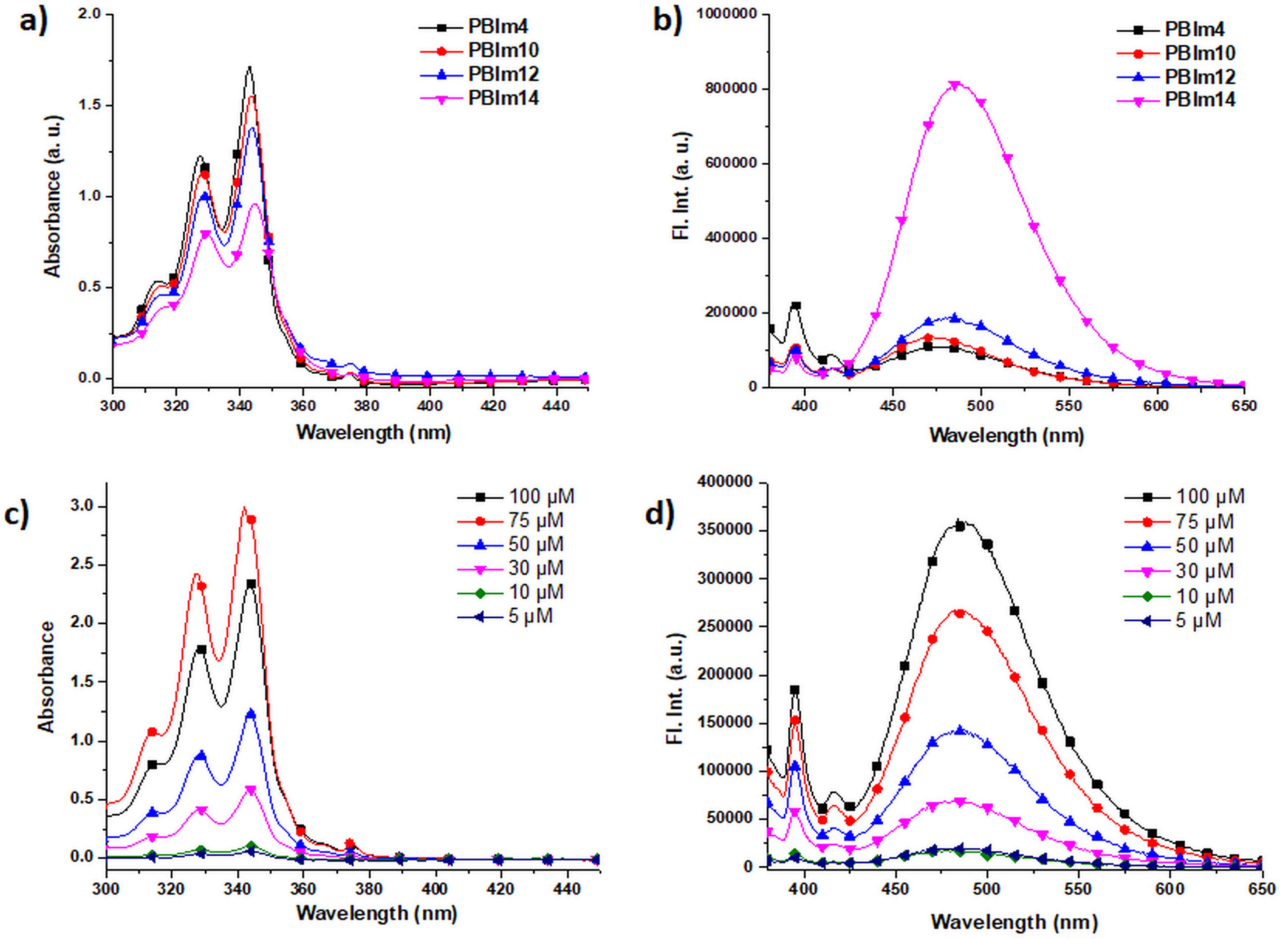
(a) Absorption and (b) emission spectra of **PBIm*N*** (50 µM) derivatives in buffer. (c) Absorption and (d) emission spectra of **PBIm12** at different concentrations in buffer.

**Figure 3:**
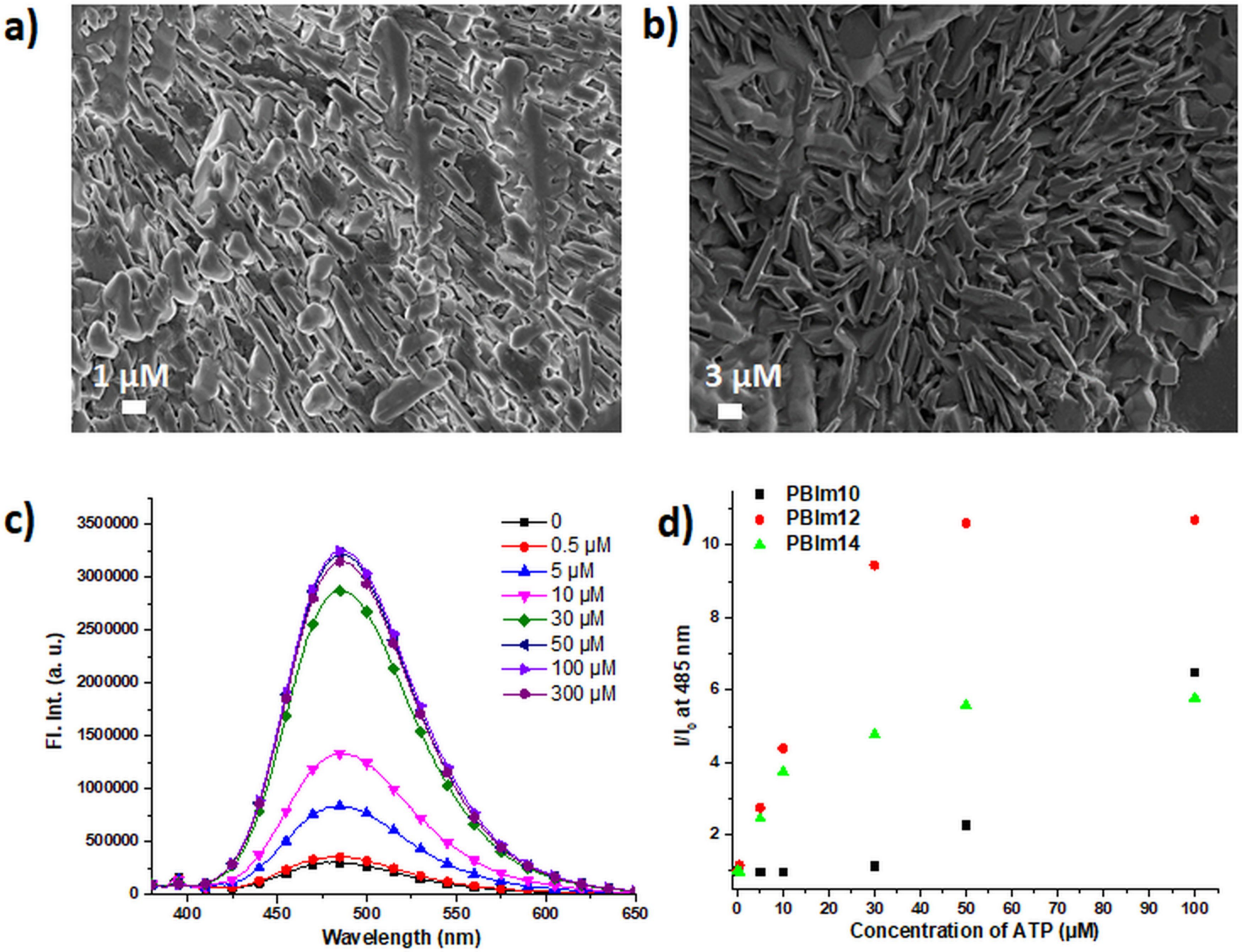
FESEM images of **PBIm12** (a) without and (b) with ATP. (c) Emission spectral changes of **PBIm12** (75 µM) in buffer upon addition of ATP. (d) Comparison plot for *I*/*I*_0_ of **PBIm10** (100 µM), **PBIm12** (75 µM) and **PBIm14** (30 µM) upon addition of different amounts of ATP.

The self-assembly properties of the four **PBIm*N*** derivatives in aqueous buffer (5 mM tris-HCl, 10 mM NaCl, water/DMSO 99.5:0.5, pH 7.4) were investigated using a combination of absorption and emission spectral studies, dynamic light scattering (DLS) and field emission scanning electron microscopy (FESEM). In a “good solvent” such as DMSO, in which all **PBIm*N***s were readily soluble, almost identical absorption (λ_max_ = 347 nm) and emission (λ_max_ = 395 nm) spectra were observed (Supporting Information File 1, Figure S1). In comparison, in aqueous buffer, certain differences existed between the butyl derivative **PBIm4** and the three longer chain derivatives **PBIm10**, **PBIm12** and **PBIm14**, hinting at the possible self-assembly behavior of the latter three molecules. In order to investigate in a detailed manner, we first carried out absorption measurements at different concentrations (Figure 2a, Supporting Information File 1, Figures S2a and S3a). All derivatives displayed three absorption peaks around 314–316 nm, 327–329 nm and 343–345 nm. These peaks were assigned to 0–0 and its vibronic replicas related to an S_0_–S_2_ transition in the pyrene derivatives [64]. The concentration dependence of the *A*_0–0_/*A*_0–1_ ratio (*A*_0–0_ and *A*_0–1_ are the absorbance of the 0–0 and 0–1 vibronic bands at 343–345 nm and 327–329 nm, respectively) generally indicates a possible mode of organization of the pyrene chromophores upon self-assembly [63]. It was found that in the concentration range of 5–50 µM [65], although a decrease in this ratio was observed for **PBIm4**; much more pronounced reductions were noticed for the *N* ≥ 10 derivatives (Figure 2a and Supporting Information File 1, Table S1), with the highest change being exhibited by **PBIm14**. These observations suggested that in going to higher concentrations, there was a gradual increase in the aggregation of the longer chain derivatives and in the aggregates, the pyrene chromophores had ground state interactions and were possibly stacked in co-facial manner as found in H-aggregates [63]. The aggregate formation was further supported by FESEM images (Figure 3a and Supporting Information File 1, Figure S7) which revealed the formation of elongated nano-objects for **PBIm10**, **PBIm12** and **PBIm14**. The existence of the aggregates was also revealed by the DLS measurements. The three *N* ≥10 derivatives exhibited nanometer size aggregates (Table 1) whereas **PBIm4** did not exhibit any aggregate formation.

**Table 1:** DLS data for the average size and zeta potential (ζ) of the self-assembled nanoaggregates of **PBIm10**, **PBIm12** and **PBIm14** before and after ATP addition in aqueous buffer (5 mM tris-HCl, 10 mM NaCl, water/DMSO, 99.5:0.5, pH 7.4).

PBIm*N* derivative	Size (nm) w/o- ATP	Size (nm) w/- ATP (50 µM)	ζ (mV) w/o- ATP	ζ (mV) w/- ATP (50 µM)

**PBIm10** (150 µM)	619.5	1582	3.45	–10.1
**PBIm12** (75 µM)	689.2	1449	2.41	–8.02
**PBIm14** (50 µM)	621.3	816	14	–6.53

The emission spectra recorded in aqueous buffer (Figure 2b) also showed a significant difference between **PBIm4** and the three amphiphilic derivatives. For example, the amphiphilic derivatives exhibited structured emission bands around 376 nm, 396 nm and 417 nm as well as a broad structureless band centered around 480–490 nm (**PBIm10**: 481 nm, **PBIm12:** 485 nm and **PBIm14**: 490 nm). These were assigned to the characteristic monomeric and excimeric emissions of pyrene, respectively [58]. The intensity of the excimeric emission was found be higher with 365 nm excitation (Supporting Information File 1, Figure S5; see Figure S6 for the excitation spectra) and hence this wavelength was used for all the subsequent studies. In comparison, **PBIm4** displayed a weaker broad band at 475 nm along with comparatively more intense monomeric bands. Since no aggregate was observed from the DLS studies in the studied concentration range, it meant that the excimeric emission presumably resulted from small clusters of **PBIm4**. Time-correlated single photon counting (TCSPC) measurements revealed ≈80 ns (Supporting Information File 1, Table S2) lifetimes for the 395 nm monomeric peak in DMSO for all the four derivatives and these values correlated well with the reported lifetimes for monomeric pyrenes [66–67]. In aqueous buffer, the lifetime of the 395 nm band decreased (≈15–35 ns, Supporting Information File 1, Table S3) probably owing to the increment in the solvent polarity [68]. The average lifetime values (τ_avg_) of the excimeric bands of **PBIm10**, **PBIm12** and **PBIm14** were found to be 33.97 ns (481 nm), 34.44 ns (485 nm) and 33.27 ns (490 nm), respectively (Supporting Information File 1, Table S3). The large Stokes shifts and the absence of vibronic features are typical characteristics of excimeric emission of pyrene [69–70].

### ATP binding studies

The aggregates with *N* ≥ 10 exhibited positive zeta potential (ζ) values under the studied conditions (Table 1) suggesting that they possessed surface exposed positive charges. Based on this, we envisaged that they could be potential ATP binders in aqueous buffer. Indeed, upon addition of ATP, zeta potential (ζ) values were significantly reduced and eventually became negative (Table 1) indicating a strong interaction of the negatively charged ATP with the aggregates. An appreciable increase in the size of the nanoaggregates was also observed upon addition of ATP (Table 1) pointing to the formation of larger co-aggregates. Furthermore, a visible change in morphology for the co-aggregates was observed from FESEM images (Figure 3b). All these results, taken together, indicated that the nanoaggregates were able to recognize ATP in a multivalent fashion leading to the formation of larger co-aggregates. On the contrary, **PBIm4** did not show the formation of aggregates even after the addition of ATP.

The most interesting feature of the ATP binding was the remarkable increase in the excimeric emission of the nano-aggregates (Figure 3c and Supporting Information File 1, Figure S8b and S8c). The sensitivity of the detection was correlated to the *I*/*I*_0_ ratio (*I* and *I*_0_ are the emission intensity of the excimeric bands with and without ATP). **PBIm12** (75 µM) displayed the best sensitivity among the three amphiphilic derivatives having a significantly enhanced *I*/*I*_0_ value of 10.6 upon 50 µM ATP addition whereas for **PBIm10** (100 µM) and **PBIm14** (30 µM) the values were 2.3 and 5.8, respectively (Figure 3d). It is noteworthy to mention here that for each of the amphiphiles, the best response was obtained at an optimum concentration of the amphiphiles. For example, both at a concentration lower and higher than 75 µM, **PBIm12** exhibited lower responses (Supporting Information File 1, Figure S9). In terms of the lower limit of detection, ATP was detected at 500 nM concentration by the **PBIm12** aggregates. A noticeable increase in the average lifetime (τ_avg_) of the excimeric band was observed after ATP addition (Supporting Information File 1, Table S4). For example, in case of **PBIm12** (75 µM), it increased from 34.44 ns to 41.99 ns upon ATP (50 µM) addition (Supporting Information File 1, Table S4). ATP also induced substantial changes in the absorption spectra for the amphiphilic derivatives (Supporting Information File 1, Figure S10b and 10c). The absorption maxima red shifted slightly and increased scattering was observed at higher amounts of ATP. The *A*_0–0_/*A*_0–1_ values were also found to decrease upon formation of the co-aggregates with ATP (Supporting Information File 1, Table S5).

The small-chain analogue, **PBIm4** (100 µM), on the contrary, showed only nominal changes in its monomeric or excimeric emission upon ATP addition (Supporting Information File 1, Figure S8a). It has already been pointed out that under these conditions, **PBIm4** was mainly present in monomeric form along with some proportions of small clusters. Previous literature reports have shown that for “molecular” imidazolium-based sensors, typically four positive charges [46,54,56] were necessary to accomplish ATP detection in the micromolar concentration range. The lack of an ATP detection capability of **PBIm4**, therefore, clearly illustrated the point that under these conditions, ATP binding was difficult for a receptor with two positive charges either in “molecular” form or as clusters and further signified the requirement of a multivalent organization of the charges as provided by the aggregates.

### Selectivity in the ATP detection

**Figure 4:**
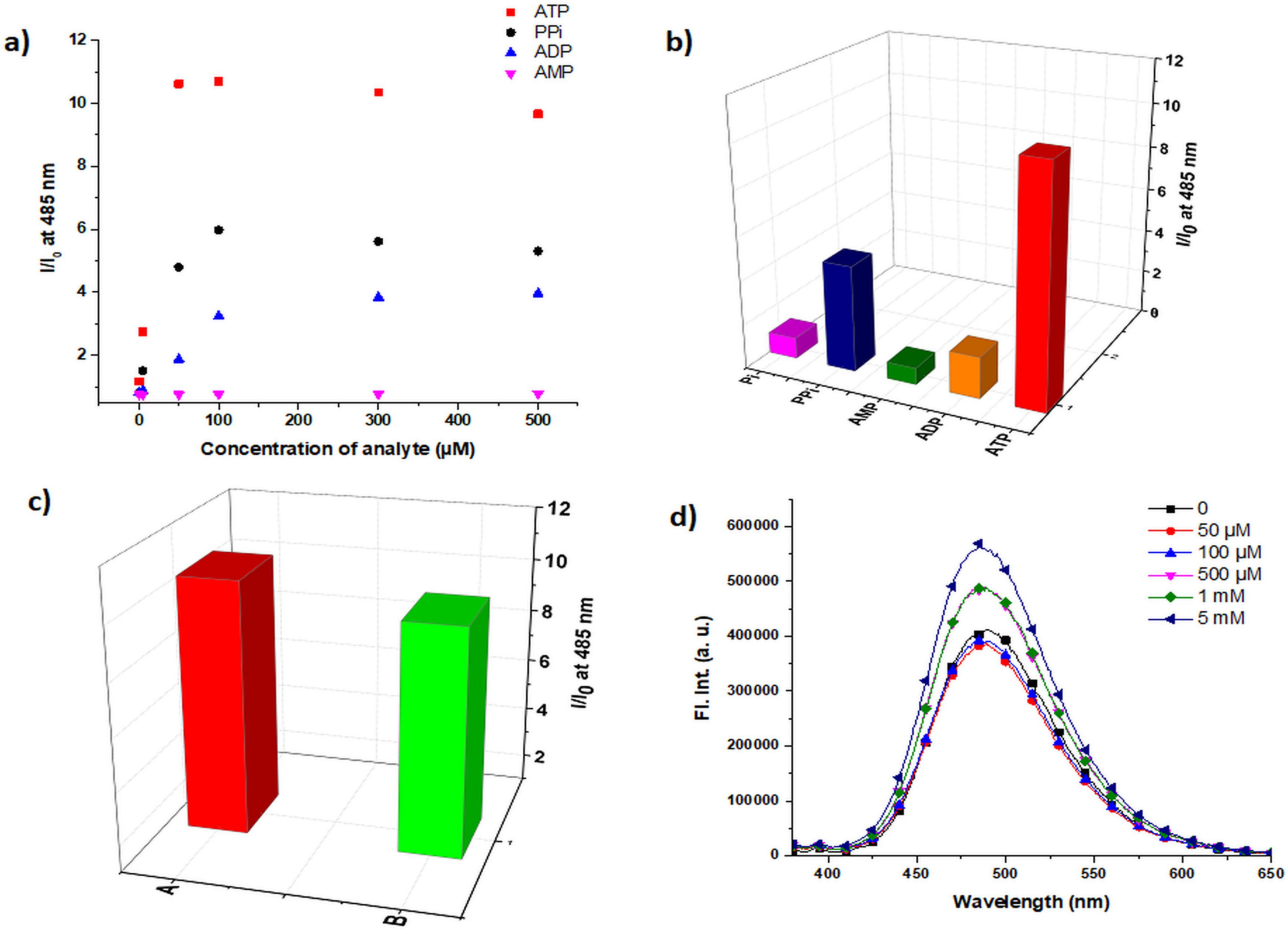
a) Emission changes of **PBIm12** (75 µM) upon the addition of ATP, ADP, AMP and PPi in buffer. Bar diagram comparing the *I*/*I*_0_ values (485 nm) of **PBIm12** (75 µM); b) upon addition of ATP, ADP, AMP, PPi and Pi in a buffer (each at 50 µM) and c) with only ATP (50 µM) (**A**) and a mixture of ATP, ADP, AMP, PPi and Pi (50 µM of each) (**B**) in buffer. d) Emission spectra of **PBIm12** (100 μM) upon the addition of ATP in a buffer containing 150 mM NaCl.

One of the key issues of a sensor design is the selectivity for an analyte in comparison to other interfering analytes. Therefore, we carried out selectivity studies with the multivalent sensors for ATP over the other adenosine phosphates ADP and AMP, PPi and inorganic phosphate (Pi). We employed **PBIm12** (75 µM) for this purpose as it exhibited the best sensitivity in the detection of ATP. A significant selectivity was noticed for ATP over ADP and AMP, PPi and Pi (Figure 4a and Figure 4b). For example, the *I*/*I*_0_ value at 485 nm for **PBIm12** (75 µM) increased to 10.62 upon ATP addition (50 µM), whereas for PPi (50 µM) and ADP (50 µM), these values were 4.8 and 1.87, respectively (Figure 4b). Practically no increments were observed for AMP (50 µM) and Pi (50 µM) (Supporting Information File 1, Figure S11a and Figure 4b). As the multivalent binding is predominantly through electrostatic interactions, it was not surprising to see a selectivity towards ATP (3 negative charges) over ADP and AMP (2 and 1 negative charge(s), respectively) at physiological pH. However, it was intriguing to note the selectivity over PPi which had four negative charges. It is likely that in the ATP induced co-assemblies, there is a more compact arrangement of the stacked pyrene chromophores and this restriction of internal motion might have resulted in a higher excimer intensity [71]. In order to see whether the presence of other phosphate analytes interfered in ATP detection, fluorescence enhancements of **PBIm12** (75 µM), for only ATP (50 µM) and ATP (50 µM) in the presence of a mixture of ADP/AMP/PPi/Pi (50 µM of each analyte) were compared. As can be seen from Figure 4c, even in the presence of four other phosphate analytes with a total concentration of 200 µM, the *I*/*I*_0_ ratio only showed a marginal decrease compared to that obtained in the presence of only ATP. This experiment thus clearly showed that the selectivity towards ATP was retained even in the presence of other phosphates. **PBIm12** (75 µM) was also able to discriminate ATP from GTP up to 30 µM concentration (Supporting Information File 1, Figure S13b). The response of **PBIm12** was also examined in the presence of many other anions such as iodide, bromide, fluoride, acetate, bicarbonate, sulfate, etc. and all of them exhibited only negligible changes in the excimeric emission (Supporting Information File 1, Figure S14).

### ATP detection in the presence of 150 mM NaCl

In order to demonstrate the practical utility of the sensors, ATP binding studies were investigated in a highly competitive medium, i.e., a buffer containing 150 mM NaCl. In a medium of such a high ionic strength, the binding affinity typically gets diminished by electrostatic screening [72]. Although the response was comparatively much lower than that observed in the plain buffer, ATP detection was possible in the concentration range of 200 µM to 5 mM using **PBIm12** (100 µM, Figure 4d) and this covered the typical concentration values of ATP in cell (3–5 mM) [73].

## Conclusion

In conclusion, we present here the synthesis, self-assembly and phosphate binding properties of four imidazolium-based receptors **PBIm*N*** (*N* = 4, 10, 12, 14). The derivatives with N ≥ 10 chains were amphiphilic in nature and underwent self-assembly in aqueous buffer to produce nano-assemblies that displayed excimeric emission from the stacked pyrene chromophores. The positive surface potential values of the nano-assemblies allowed them to bind to ATP which was accompanied by a noticeable increase in the intensity of the excimeric emission. Furthermore, the multivalent assemblies displayed a high-level selectivity towards ATP over other adenosine phosphates (ADP and AMP) and pyrophosphate (PPi). The detection of ATP was also possible in the presence of 150 mM NaCl. The modular nature of the sensing platform provided by the multivalent nano-assemblies of the bisimidazolium amphiphiles can in principle be extended for the detection of other biologically important analytes through a structural modulation of the amphiphiles and the studies along this direction are currently in progress in our group.

## Experimental

### Materials and methods

All starting materials were purchased from commercially available sources and used without further purification. All the nucleotides, 1-pyrenecarboxaldehyde, sodium borohydride, sodium hydride, phosphorus tribromide and 1-bromobutane, 1-bromodecane, 1-bromododecane and 1-bromotetradecane were purchased from Sigma-Aldrich. Imidazole, dibromomethane and tris(hydroxymethyl)aminomethane were purchased from TCI chemicals. Sodium chloride, 28% ammonia solution and all the solvents were purchased from Merck. Milli-Q water was used for all the experiments. UV–vis spectroscopic measurements were carried out in Agilent Cary 8454 spectrophotometer. Emission spectroscopic measurements were carried out in a Horiba Fluoromax 4 spectrofluorometer. Images were taken under a 365 nm UV lamp. DLS and zeta potential measurements were carried out using a Malvern Zetasizer NanoZS. A Horiba Jobin Yvon Fluorocube instrument fitted with a 340 nm diode laser excitation source (with a temporal resolution of 70 ps) was used for the time-resolved fluorescence experiments applying the time correlated single photon counting (TCSPC) method. ^1^H and ^13^C NMR were performed on Jeol 400 MHz and Bruker 500 MHz spectrometers. Mass spectra were recorded in a Bruker mass spectrometer. SEM images was recorded by using the instrument CARL ZEISS (model SUPRA 55VP).

### Preparation of solutions

#### A. Preparation of PBImN solutions

Initially, stock solutions of **PBIm*N***s (*N* = 4, 10, 12, 14) were prepared by dissolving the solid powders in spectroscopic grade dimethyl sulfoxide (DMSO). These concentrated DMSO solutions were then diluted to 5 mM tris-HCl buffer in 10 mM NaCl made in Milli-Q water to get the desired solutions having 1 or 0.5% DMSO in the final DMSO fraction. Solutions of **PBIm4** in the buffer were equilibrated for 10 min whereas the solutions of **PBIm10**, **PBIm12** and **PBIm14** were equilibrated for 15 min.

#### B. Preparation of stock solutions of nucleotides

Stock solutions of nucleotides (10 mM) were prepared by dissolving the required amounts of them in Milli-Q water.

### Titration procedures

During the fluorometric titration, in every set of titrations a fresh solution of **PBIm*N*** was prepared and equilibrated for 15 min and then the spectra were recorded.

### FESEM sample preparation

Aqueous buffer solutions of **PBIm*N***s (*N* = 10, 12 and 14) were first stabilized for 15 min and then 5 µL of these solutions were drop casted on glass cover slips. The samples were dried in air overnight and then under high vacuum for 8–10 hours. Finally, the morphologies of the samples were recorded in a FESEM instrument. Morphologies of ATP-added **PBIm*N***s samples were recorded after addition of ATP to the **PBIm*N***s solutions followed by drying the drop casted solutions of ATP-added **PBIm*N***s on glass cover slips in a similar way.

## Supporting Information

File 1 Synthetic procedures, additional spectroscopic, microscopic and dynamic light scattering data (figures and tables) and NMR characterization.

## References

[1] Busschaert N, Caltagirone C, Van Rossom W, Gale P A (2015). Chem Rev.

[2] Flood A H (2016). Beilstein J Org Chem.

[3] Wu J, Zou Y, Li C, Sicking W, Piantanida I, Yi T, Schmuck C (2012). J Am Chem Soc.

[4] Maity D, Schmuck C (2016). Chem – Eur J.

[5] Maeda H (2008). Chem – Eur J.

[6] Zhang Z, Kim D S, Lin C-Y, Zhang H, Lammer A D, Lynch V M, Popov I, Miljanić O Š, Anslyn E V, Sessler J L (2015). J Am Chem Soc.

[7] You L, Zha D, Anslyn E V (2015). Chem Rev.

[8] Mammen M, Choi S-K, Whitesides G M (1998). Angew Chem, Int Ed.

[9] Badjić J D, Cantrill S J, Stoddart J F (2004). J Am Chem Soc.

[10] Crespo-Biel O, Lim C W, Ravoo B J, Reinhoudt D N, Huskens J (2006). J Am Chem Soc.

[11] Badjić J D, Nelson A, Cantrill S J, Turnbull W B, Stoddart J F (2005). Acc Chem Res.

[12] Lee Y C, Lee R T (1995). Acc Chem Res.

[13] Lin B, Qing X, Liao J, Zhuo K (2020). Cells.

[14] Krachler A M, Ham H, Orth K (2011). Proc Natl Acad Sci U S A.

[15] Fasting C, Schalley C A, Weber M, Seitz O, Hecht S, Koksch B, Dernedde J, Graf C, Knapp E-W, Haag R (2012). Angew Chem, Int Ed.

[16] Liang J, Li K, Liu B (2013). Chem Sci.

[17] Feng X, Liu L, Wang S, Zhu D (2010). Chem Soc Rev.

[18] Rochat S, Swager T M (2013). ACS Appl Mater Interfaces.

[19] Huskens J, Prins L J, Haag R (2018). Multivalency: Concepts, Research and Applications.

[20] Dreher M R, Simnick A J, Fischer K, Smith R J, Patel A, Schmidt M, Chilkoti A (2008). J Am Chem Soc.

[21] Kingery-Wood J E, Williams K W, Sigal G B, Whitesides G M (1992). J Am Chem Soc.

[22] Drożdż W, Walczak A, Bessin Y, Gervais V, Cao X-Y, Lehn J-M, Ulrich S, Stefankiewicz A R (2018). Chem – Eur J.

[23] Rodrigo A C, Barnard A, Cooper J, Smith D K (2011). Angew Chem, Int Ed.

[24] Rajangam K, Behanna H A, Hui M J, Han X, Hulvat J F, Lomasney J W, Stupp S I (2006). Nano Lett.

[25] Xu Z, Jia S, Wang W, Yuan Z, Ravoo B J, Guo D-S (2019). Nat Chem.

[26] Sandanaraj B S, Vutukuri D R, Simard J M, Klaikherd A, Hong R, Rotello V M, Thayumanavan S (2005). J Am Chem Soc.

[27] Bhaumik S K, Patra Y S, Banerjee S (2020). Chem Commun.

[28] Knowles J R (1980). Annu Rev Biochem.

[29] Higgins C F, Hiles I D, Salmond G P C, Gill D R, Downie J A, Evans I J, Holland I B, Gray L, Buckel S D, Bell A W (1986). Nature.

[30] Yu M, Yao Y, Cui B, Sun C, Zhao X, Wang Y, Liu G, Cui H, Zeng Z (2019). ACS Appl Nano Mater.

[31] Bhowmik S, Ghosh B N, Marjomäki V, Rissanen K (2014). J Am Chem Soc.

[32] Weitz E A, Chang J Y, Rosenfield A H, Morrow E A, Pierrre V C (2013). Chem Sci.

[33] Kurishita Y, Kohira T, Ojida A, Hamachi I (2012). J Am Chem Soc.

[34] Ngo H T, Liu X, Jolliffe K A (2012). Chem Soc Rev.

[35] Hargrove A E, Nieto S, Zhang T, Sessler J L, Anslyn E V (2011). Chem Rev.

[36] Zuo X, Song S, Zhang J, Pan D, Wang L, Fan C (2007). J Am Chem Soc.

[37] Tang Z, Mallikaratchy P, Yang R, Kim Y, Zhu Z, Wang H, Tan W (2008). J Am Chem Soc.

[38] Liu Z, Chen S, Liu B, Wu J, Zhou Y, He L, Ding J, Liu J (2014). Anal Chem (Washington, DC, U S).

[39] Zhou Q, Swager T M (1995). J Am Chem Soc.

[40] Parthasarathy A, Pappas H C, Hill E H, Huang Y, Whitten D G, Schanze K S (2015). ACS Appl Mater Interfaces.

[41] Cui Q, Yang Y, Yao C, Liu R, Li L (2016). ACS Appl Mater Interfaces.

[42] Zhao Q, Zhang Z, Tang Y (2017). Chem Commun.

[43] Huang H, Tan Y, Shi J, Liang G, Zhu J-J (2010). Nanoscale.

[44] Zhong Y, Yi T (2019). J Mater Chem B.

[45] Maity D, Li M, Ehlers M, Schmuck C (2017). Chem Commun.

[46] Xu Z, Singh N J, Lim J, Pan J, Kim H N, Park S, Kim K S, Yoon J (2009). J Am Chem Soc.

[47] Li X, Guo X, Cao L, Xun Z, Wang S, Li S, Li Y, Yang G (2014). Angew Chem, Int Ed.

[48] Nishizawa S, Kato Y, Teramae N (1999). J Am Chem Soc.

[49] Noguchi T, Shiraki T, Dawn A, Tsuchiya Y, Ngoc Lien L T, Yamamoto T, Shinkai S (2012). Chem Commun.

[50] Huang L-X, Guo Q, Chen Y, Verwilst P, Son S, Wu J-B, Cao Q-Y, Kim J S (2019). Chem Commun.

[51] Xu H-R, Li K, Jiao S-Y, Pan S-L, Zeng J-R, Yu X-Q (2015). Analyst.

[52] Ma H, Yang M, Zhang C, Ma Y, Qin Y, Lei Z, Chang L, Lei L, Wang T, Yang Y (2017). J Mater Chem B.

[53] Alcalde E, Mesquida N, Vilaseca M, Alvarez-rúa C, García-Granda S (2007). Supramol Chem.

[54] Ahmed N, Shirinfar B, Geronimo I, Kim K S (2011). Org Lett.

[55] Wang D, Zhang X, He C, Duan C (2010). Org Biomol Chem.

[56] Kwon J Y, Singh N J, Kim H N, Kim S K, Kim K S, Yoon J (2004). J Am Chem Soc.

[57] Maity D, Assaf K I, Sicking W, Hirschhäuser C, Nau W M, Schmuck C (2019). Chem – Eur J.

[58] Winnik F M (1993). Chem Rev.

[59] Ge Y, Wen Y, Liu H, Lu T, Yu Y, Zhang X, Li B, Zhang S-T, Li W, Yang B (2020). J Mater Chem C.

[60] Biswas R, Naskar S, Ghosh S, Das M, Banerjee S (2020). Chem – Eur J.

[61] Kumari N, Dey N, Jha S, Bhattacharya S (2013). ACS Appl Mater Interfaces.

[62] Černochová J, Branná P, Rouchal M, Kulhánek P, Kuřitka I, Vícha R (2012). Chem – Eur J.

[63] Yang C, Mehmood F, Lam T L, Chan S L-F, Wu Y, Yeung C-S, Guan X, Li K, Chung C Y-S, Zhou C-Y (2016). Chem Sci.

[64] Haedler A T, Misslitz H, Buehlmeyer C, Albuquerque R Q, Köhler A, Schmidt H-W (2013). ChemPhysChem.

[R65] 65Above 50 µM, the ratios were not calculated since the absorbance values were high.

[66] Kalyanasundaram K, Thomas J K (1977). J Am Chem Soc.

[67] Somerharju P (2002). Chem Phys Lipids.

[68] Karpovich D S, Blanchard G J (1995). J Phys Chem.

[69] Lehrer S S (1995). Subcell Biochem.

[70] Birks J B (1970). Photophysics of aromatic Molecules.

[71] Chan C W, Smith D K (2016). Chem Commun.

[72] Jiao Q, Liu Q (1998). Anal Lett.

[73] Gribble F M, Loussouarn G, Tucker S J, Zhao C, Nichols C G, Ashcroft F M (2000). J Biol Chem.

