# Supplementary material for: Selective recognition of ATP by multivalent nano-assemblies of bisimidazolium amphiphiles through “turn-on” fluorescence response
Source: Beilstein J Org Chem. 2020 Nov 10;16:2728–38. doi: 10.3762/bjoc.16.223 (PMC7670119; doi:10.3762/bjoc.16.223)

BEILSTEIN JOURNAL OF ORGANIC CHEMISTRY

Supporting Information

for

Selective recognition of ATP by multivalent nano-assemblies of bisimidazolium amphiphiles through "turn-on" fluorescence response

Rakesh Biswas, Surya Ghosh, Shubhra Kanti Bhaumik and Supratim Banerjee

Beilstein J. Org. Chem. **2020,** *16,* 2728–2738. doi:10.3762/bjoc.16.223

Synthetic procedures, additional spectroscopic, microscopic and dynamic light scattering data (figures and tables) and NMR characterization

License and Terms: This is a supporting information file under the terms of the Creative Commons Attribution License (https://creativecommons.org/licenses/by/4.0). Please note that the reuse, redistribution and reproduction in particular requires that the authors and source are credited.
The license is subject to the *Beilstein Journal of Organic Chemistry* terms and conditions: (https://www.beilstein-journals.org/bjoc)

Table of contents **Page No.**

Synthetic procedures S03

Results and discussions S08

1. **General spectroscopic studies** S08
2. **Morphological study** S11
3. **Titration with phosphates in buffer** S11
4. **Lifetime measurements** S14
5. **Size and zeta potential measurements** S16
6. **NMR spectra** S22

Synthetic procedures:

Pyren-1-ylmethanol (2): In a 100 mL round bottom flask, **1** (600 mg, 2.60 mmol), and sodium borohydride (296 mg, 7.82 mmol) were dissolved in 30 mL ethanol and the yellow reaction mixture was refluxed for 3 h. After 3 h, it turned colorless. It was cooled to room temperature and a saturated Na_2CO_3 solution (50 mL) was added to it. After stirring for 30 min, the reaction mixture was taken in separating funnel and extracted with DCM (5 x 100 mL). The organic layers were combined, dried over anhydrous Na_2SO_4, filtered and the solvent was evaporated in a rotary evaporator. The crude product was dried under high vacuum and was purified by a silica gel column using 30% EtOAc : hexane as the eluent. Product **2** was obtained as a white solid. Yield: 561 mg (93%). **^{1}H NMR (400 MHz, $CDCl_3$): δ (ppm)** = 8.39 (d, *J* = 9.2 Hz, 1H), 8.22-8.15 (m, 4H), 8.09-8.00 (m, 4H), 5.42 (s, 2H).

1-(Bromomethyl)pyrene (3): Compound **2** (563 mg, 2.42 mmol) was dissolved in 20 mL toluene in a 50 mL two necked round bottom flask equipped with a $CaCl_2$ guard tube and cooled in an ice-salt bath. Phosphorous tribromide (19.36 mmol) was added to the solution drop wise using a syringe and the reaction mixture was stirred at 0 °C. After 10 h, the colorless reaction mixture turned into a white suspension. After this, the reaction mixture was treated slowly with a saturated Na_2CO_3 solution (30 mL) to neutralize the excess phosphorous tribromide and stirred for further 15 min at room temperature. Then the solution was taken in a separating funnel and the organic compound was extracted in toluene (4 × 100 mL). The light yellow colored organic layers were combined and was washed with water, brine and finally one more time with water. The yellow solution was dried over anhydrous Na_2SO_4, filtered and the solvent was evaporated in a rotary evaporator. Product **3** was obtained as a light yellow solid. Yield: 614 mg (86%). **^{1}H NMR (400 MHz, $CDCl_3$): δ (ppm)** = 8.39 (d, *J* = 9.2 Hz, 1H); 8.26-8.22 (m, 3H); 8.14-8.02 (m, 5H); 5.27 (s, 2H).

Di(1*H*-imidazol-1-yl)methane (5): Imidazole (**4**, 2 g, 29.4 mmol) and sodium hydride (1.4 g, 58.8 mmol) were taken in a 100 mL round bottom flask. Distilled DMF (10 mL) was added and the reaction mixture was stirred in an ice bath for 10 min. CH_2Br_2 (11.76 mmol) was then slowly added into the reaction mixture and the stirring was continued for the next 48 h at room temperature. Solvent was then evaporated and the residue was taken in 50 mL water in a 250 mL separating funnel and extracted with dichloromethane (100 mL × 5). The organic phases were combined and dried over anhydrous Na_2SO_4. Then solvent was removed to get the desired product. The amount of product was 1.22 g. Yield = 70%. **^{1}H NMR: (400 MHz, DMSO-*d*$_6$): δ (ppm)** = 7.92 (s, 2H); 7.39 (s, 2H); 6.90 (s, 2H); 6.21 (s, 2H).

BIm4: 5 (1.036 gm, 7 mmol) and 10 mL of DMF were taken in a 25 mL round bottom flask. 1-Bromobutane (0.7 mmol) was then added to it in portions at 2 h intervals with continuous stirring at 100 °C. After 12 h, the solvent was removed and the product was purified by two successive column chromatography using neutral alumina as the solid phase and 10–40% methanol/ethyl acetate as the eluent. Finally, the solvent was removed and the product was obtained as a semisolid. The amount of the product was 27 mg. Yield = 27%. **^{1}H NMR (400 MHz, DMSO-*d*$_6$): δ (ppm)** = 9.61 (s, 1H), 8.08 (s, 1H), 8.04 (s, 1H), 7.86 (s, 1H), 7.55 (s, 1H), 6.98 (s, 1H), 6.53 (s, 2H), 4.20 (t, J = 7.2 Hz, 2H), 1.77 (quint, J = 7.3 Hz, 2H), 1.24 (m, 2H), 0.88 (m, 3H). **^{13}C NMR (125 MHz, DMSO-*d*$_6$): δ (ppm)** = 137.88, 136.55, 129.67, 123.05, 121.87, 119.35, 56.74, 49.19, 48.93, 31.10, 30.49, 29.07, 25.12, 21.87, 18.78, 13.83, 13.28. **HRMS (ESI):** m/z calculated for $C_{11}H_{17}N_4^+$ 205.1448; found 205.1435.

BIm10: 5 (602 mg, 4.07 mmol), 1-bromodecane (1.35 mmol) and 10 mL of DMF were taken in a 25 mL round bottom flask and stirred at 100 °C for 24 hours. Solvent was then removed and the product was purified by two successive column chromatography using neutral alumina as the solid phase and 20–50% methanol/ethyl acetate as the eluent. Finally, the solvent was removed and the product was obtained as a semisolid. The amount of the product was 298 mg.

Yield = 60%. **^{1}H NMR: (400 MHz, $CDCl_3$): δ (ppm)** = 10.31 (s, 1H); 7.71 (s, 1H); 7.43 (s, 1H); 7.16 (s, 1H); 7.01 (s, 1H); 6.73 (s, 2H); 4.17 (t, 2H, *J* = 7.6 Hz); 1.83 (m, 2H); 1.29-1.23 (m, 14H); 0.85 (t, 3H, *J* = 7.2 Hz). **^{13}C NMR: (100 MHz, $CDCl_3$): δ (ppm) =** 138.08, 130.27, 122.06, 121.99, 121.90, 119.54, 57.65, 50.46, 31.93, 30.05, 29.53, 29.43, 29.32, 29.00, 26.31, 22.75, 14.18. **HRMS (ESI):** m/z calculated for $C_{17}H_{29}N_4^+$ 289.2387; found 289.2396.

BIm12: 5 (600 mg, 4.04 mmol), 1-bromododecane (0.320 ml, 1.34 mmol,) and 10 mL of DMF were taken in a 25 mL round bottom flask and stirred at 100 °C for 24 hours. Solvent was then removed and the product was purified by two successive column chromatography using neutral alumina as the solid phase and 20–50% methanol/ethyl acetate as the eluent. Finally, the solvent was removed and the product was obtained as a semisolid. The amount of the product was 329 mg. Yield = 62%. **^{1}H NMR: (400 MHz, DMSO- *d*$_6$): δ (ppm)** =10.05 (s, 1H); 8.08 (d, *J* = 7.6 Hz, 2H); 7.84 (s, 1H); 7.56 (s, 1H); 6.95 (s, 1H); 6.59 (s, 2H); 4.18 (t, *J* = 6.8 Hz, 2H); 1.30-1.15 (m, 18H); 0.84 (t, *J* = 6.8 Hz, 3H). **^{13}C NMR: (100 MHz, DMSO-*d*$_6$): δ (ppm)** = 173.82; 137.93; 137.31; 129.56; 122.93; 121.91; 119.35; 56.63; 49.11; 31.30; 29.02; 28.93; 28.83; 28.72; 28.35; 25.49; 23.82; 22.10; 13.93. **HRMS (ESI):** m/z calculated for $C_{19}H_{33}N_4^+$: 317.2700; found 317.2692.

BIm14: 5 (600 mg, 4.04 mmol), 1-bromotetradecane (0.4 ml, 1.346 mmol) and 10 mL of DMF were taken in a 25 mL round bottom flask and stirred at 100 °C for 24 hours. Solvent was then removed and the product was purified by two successive column chromatography using neutral alumina as the solid phase and 20–50% methanol/ ethyl acetate as the eluent. Finally, the solvent was removed and the product was obtained as a semisolid. The amount of the product obtained was 399 mg. Yield = 70%. **^{1}H NMR: (400 MHz, $CDCl_3$): δ (ppm)** = 10.47 (s, 1H); 8.07 (s, 1H); 7.81 (s, 1H); 7.45 (s, 1H); 7.16 (s, 1H); 6.96 (s, 1H); 6.77 (s, 2H); 4.15 (t, *J* = 7.2 Hz, 2H); 1.96 (m, 2H); 1.27-1.22 (m, 22 H); 0.85 (t, *J* = 7.2 Hz, 3H). **^{13}C NMR: (100 MHz, DMSO-*d*$_6$): δ (ppm)** = 173.19; 137.90; 136.82; 129.69; 123.05; 121.90; 119.33; 79.22; 56.81;

49.20; 31.33; 29.16; 29.05; 28.96; 28.85; 28.74; 28.37; 25.51; 22.66; 22.13; 13.98. **HRMS (ESI):** m/z calculated for $C_{21}H_{37}N_4^+$: 345.3013; found 345.3013.

PBIm*N* (*N* = 4, 10, 12, 14): In a typical procedure, **BIm*N*** (*N* = 4, 10, 12 and 14; 1 equiv.), **3** (1.5 equiv.) and 1 mL of toluene were taken in a 5 mL round bottom flask and stirred at reflux condition for 48 hours. The light-yellow precipitates were filtered and washed with toluene for five times. The residues were finally dried under vacuum.

PBIm4: From 25 mg of **BIm4,** 26 mg of **PBIm4** was obtained as a light-yellow powder. Yield = 50%. **^{1}H NMR (400 MHz, DMSO-*d$_6$*): δ (ppm)** = 9.59 (s, 1H); 9.54 (s, 1H); 8.48-8.35 (m, 4H); 8.28-8.11 (m, 5H), 8.04 (s, 1H), 8.00 (s, 1H), 7.89 (s, 1H), 6.68 (s, 2H), 6.30 (s, 2H), 4.21 (t, *J* = 7.2 Hz, 2H), 1.80-1.74 (m, 2H), 1.22-1.15 (m, 2H), 0.91-0.84 (m, 3H). **^{13}C NMR (125 MHz, DMSO-*d$_6$*): δ (ppm)** = 138.27, 138.0, 132.20, 131.20, 130.66, 129.42 129.31, 128.87, 127.78, 127.27, 127.19, 126.63, 126.40, 125.75, 124.64, 124.16, 124.06, 123.66, 122.96, 122.69, 58.88, 50.82, 49.72, 49.53, 31.50, 30.92, 29.66, 19.33, 14.22, 13.67. **HRMS (ESI):** m/z calculated for $C_{28}H_{28}N_4^{2+}$ (M-Na): 221.6101; found: 221.6045.

PBIm10: From 60 mg of **BIm10,** 68 mg of **PBIm10** was obtained as a light-yellow powder. Yield = 63%. **^{1}H NMR (400 MHz, DMSO-*d$_6$*): δ(ppm)** = 9.68 (s, 1H); 9.61 (s, 1H); 8.51-8.49 (d, *J* = 8 Hz, 1H,); 8.41- 8.34 (m, 4H); 8.30- 8.22 (m, 3H); 8.16 -8.12 (m, 2H); 8.09 (s, 1H); 8.01 (s, 1H); 7.91 (s, 1H); 6.68 (s, 2H); 6.32 (s, 2H); 4.20 (t, *J* = 8 Hz, 2H); 1.77 (m, 2H); 1.35-1.15 (m, 14H); 0.84 (t, *J* = 8 Hz, 3H). **^{13}C NMR (100 MHz, DMSO-*d$_6$*): δ (ppm)** = 137.79; 137.90; 131.67; 130.68; 130.14; 128.80; 128.39; 128.31; 127.26; 126.71; 126.08; 125.87; 125.23; 124.12; 123.65; 123.49; 123.13; 122.15; 58.17; 50.46; 49.28; 31.27; 29.10; 28.89; 28.82; 28.66; 28.38; 25.46; 22.09 **HRMS (ESI):** m/z calculated for $C_{34}H_{40}N_4^{2+}$ (M+H$^+$): 252.1621; found 252.1605.

PBIm12: From 90 mg of **BIm12,** 122 mg of **PBIm12** was obtained as a light yellow compound. Yield = 77%. **^{1}H NMR: (400 MHz, DMSO- *d$_6$*): δ (ppm)** = 9.64 (s, 1H); 9.57 (s, 1H); 8.51-

8.48 (m, 1H); 8.41- 8.34 (m, 4H); 8.29- 820 (m, 3H); 8.17- 8.13 (m, 2H); 8.06 (s, 1H); 8.00 (s, 1H); 7.89 (s, 1H); 4.19 (t, *J* = 8 Hz, 2H); 1.76 (m, 2H); 1.21 (m, 17H); 0.83 (t, *J* = 8 Hz, 3H). **^{13}C NMR: (100 MHz, DMSO-*d*$_6$):** δ (ppm) = 137.79; 137.49; 131.76; 130.71; 130.15; 127.27; 126.75; 128.39; 126.75; 126.12; 125.89; 125.25; 123.74; 123.68; 123.52; 123.16; 122.45; 122.17; 58.22; 50.48; 49.30; 31.29; 29.11; 29.01; 28.95; 28.83; 28.71; 28.39; 25.47; 22.10. **HRMS (ESI):** m/z calculated for $C_{36}H_{44}N_4^{2+}$ (M+H$^+$): 531.3477; found 531.3441.

PBIm14: From 53 mg of **BIm14,** 63 mg of **PBIm14** was obtained as a light-yellow compound. Yield = 70%. **^{1}H NMR: (400 MHz, DMSO-*d*$_6$): δ (ppm)** = 9.70 (s, 1H); 9.63 (s, 1H); 8.51-8.49 (d, *J* = 8 Hz, 1H); 8.41-8.34 (m, 4H); 8.29-8.21 (m, 3H); 8.16-8.13 (m, 2H); 8.10 (s, 1H); 8.01 (s, 1H); 7.90 (s, 1H); 4.20 (t, *J* = 8 Hz, 2H); 1.77 (m, 2H); 0.83 (t, *J* = 8 Hz, 3H) **^{13}C NMR: (100 MHz, DMSO-*d*$_6$): δ (ppm)** = 137.80; 137.90; 131.66; 130.67; 130.14; 128.79; 128.37; 128.30; 127.25; 126.71; 126.07; 125.85; 125.22; 124.11; 123.66; 123.47; 123.11; 122.43; 122.15; 58.15; 49.27; 31.28; 29.09; 29.04; 29.01; 28.95; 28.82; 28.70; 28.39; 25.47; 22.08; 13.96. **HRMS (ESI):** m/z calculated for $C_{38}H_{48}N_4^{2+}$ (M+H$^+$): 559.3790; found 559.3785.

Results and discussions:

1. General spectroscopic studies:

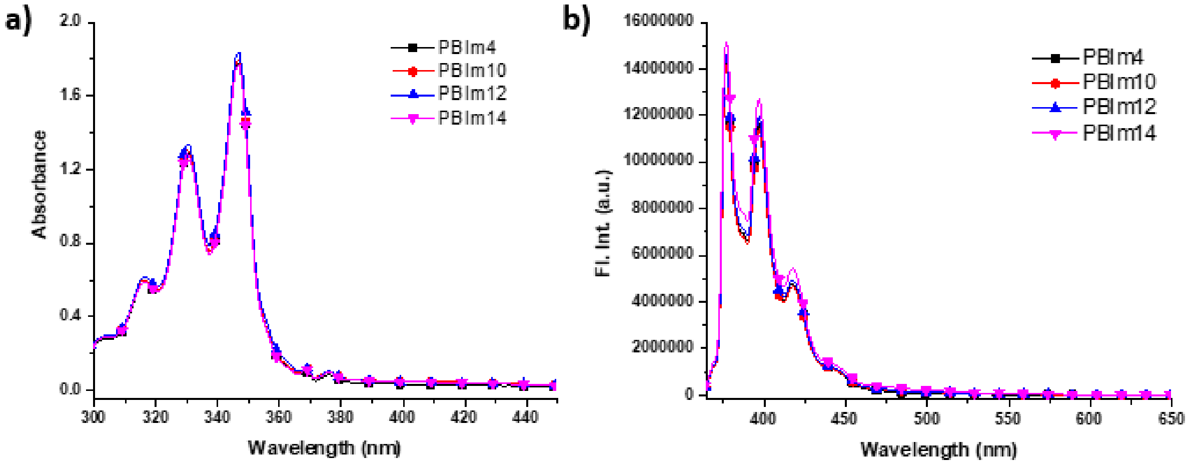

Figure S1. (a) Absorption spectra and (b) emission spectra of **PBIm4**, **PBIm10**, **PBIm12** and **PBIm14** (50 µM of each) in DMSO (λ_{ex} = 350 nm).

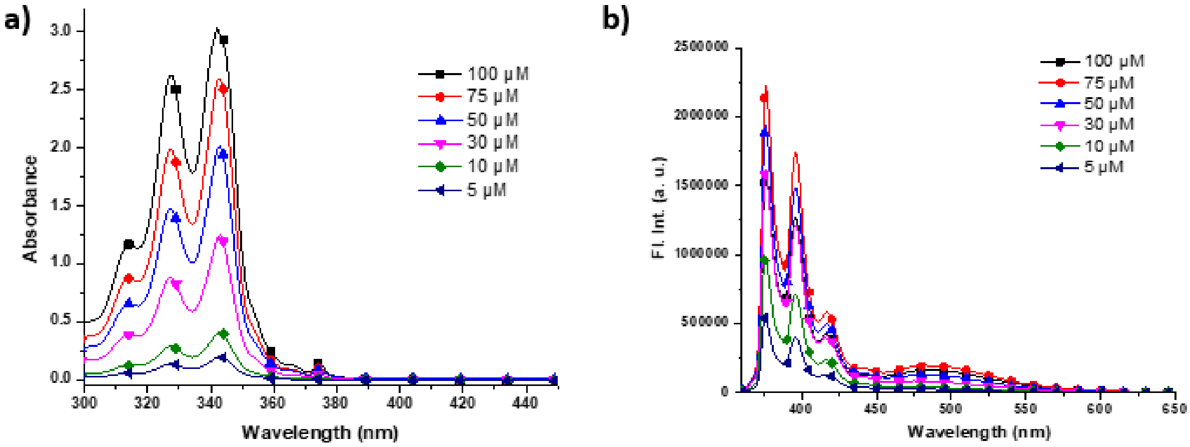

Figure S2. (a) Absorption spectra and (b) emission spectra (λ_{ex} = 350 nm) of **PBIm4** at different concentration in aqueous buffer (5 mM tris in 10 mM NaCl, pH 7.4).

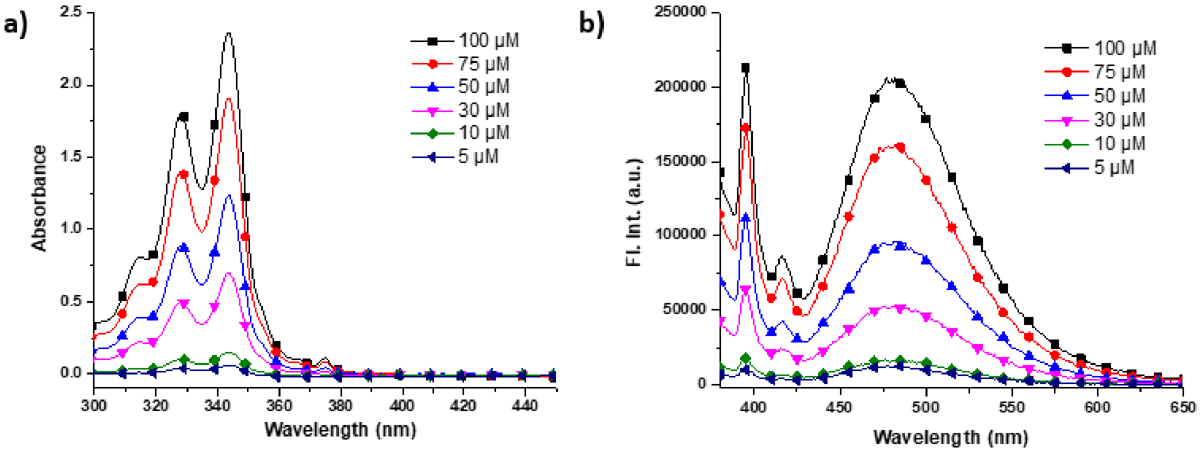

Figure S3. (a) Absorption spectra and (b) emission spectra (λ_{ex} = 365 nm) of **PBIm10** at different concentration in aqueous buffer (5 mM tris in 10 mM NaCl, pH 7.4).

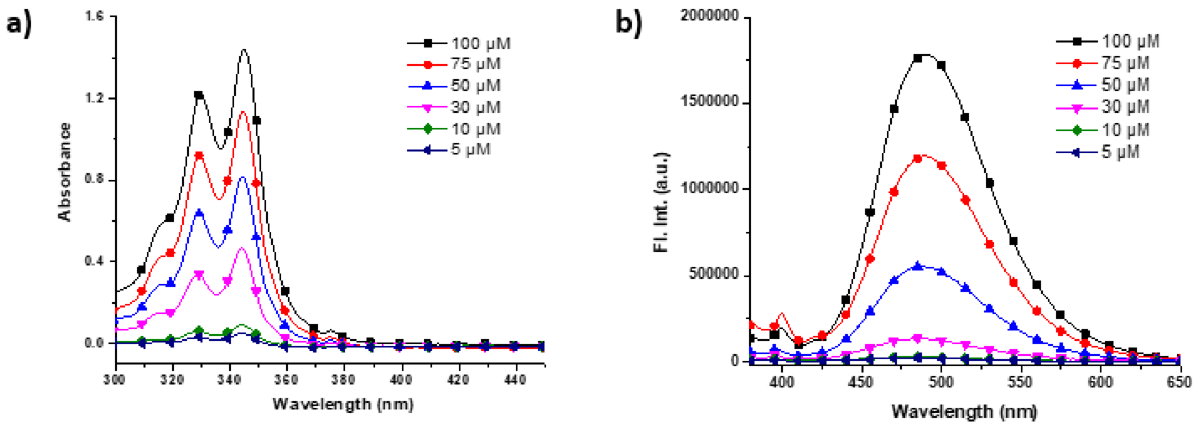

Figure S4. (a) Absorption spectra and (b) emission spectra (λ_{ex} = 365 nm) of **PBIm14** at different concentration in aqueous buffer (5 mM tris in 10 mM NaCl, pH 7.4).

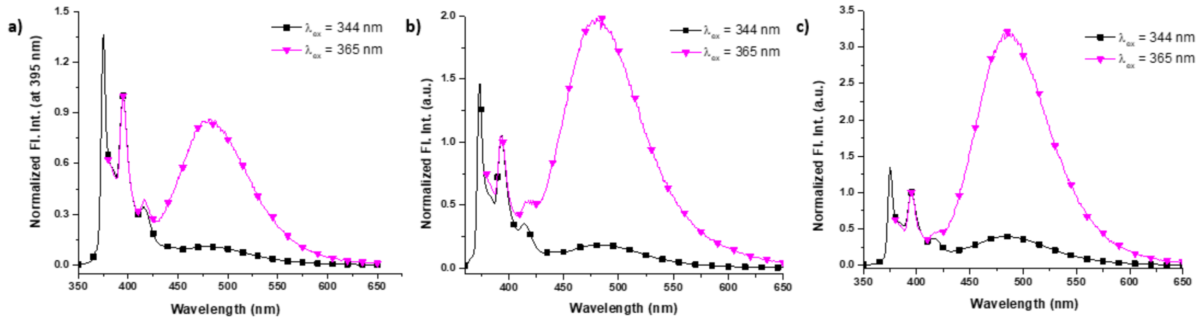

Figure S5. Emission spectra of (a) **PBIm10** (50 µM), (b) **PBIm12** (75 µM) and (c) **PBIm14** (30 µM) upon excited with different wavelengths of light in aqueous buffer (5 mM tris in 10 mM NaCl, pH 7.4).

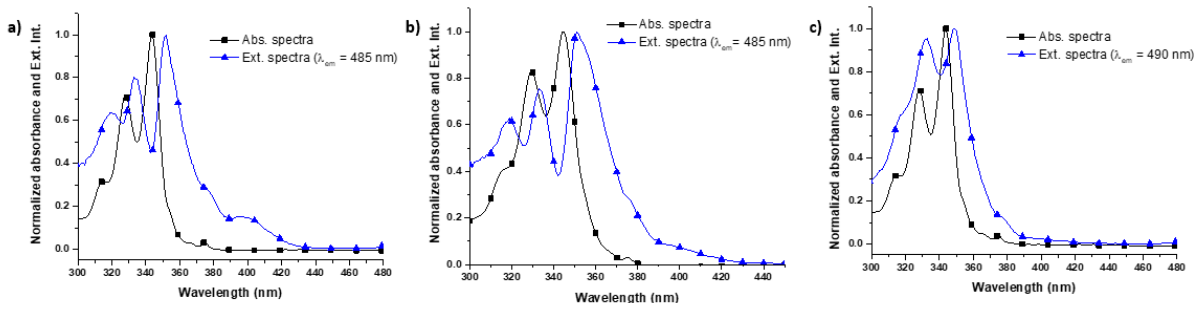

Figure S6. Absorption spectra excitation spectra of (a) **PBIm10** (50 µM), (b) **PBIm12** (75 µM) and (c) **PBIm14** (30 µM) in aqueous buffer (5 mM tris in 10 mM NaCl, pH 7.4).

Table S1. $A_{0\text{-}0}/A_{0\text{-}1}$ ratios for **PBIm4, PBIm10, PBIm12** and **PBIm14** at different concentrations in aqueous buffer (5 mM tris in 10 mM NaCl, pH 7.4).

**PBIm*N* derivatives (in buffer)**	**$A_{0\text{-}0}/A_{0\text{-}1}$ at 50 μM**	**$A_{0\text{-}0}/A_{0\text{-}1}$ at 30 μM**	**$A_{0\text{-}0}/A_{0\text{-}1}$ at 10 μM**	**$A_{0\text{-}0}/A_{0\text{-}1}$ at 5 μM**
**PBIm4**	1.37	1.426	1.452	1.467
**PBIm10**	1.385	1.396	1.49	1.63
**PBIm12**	1.395	1.40	1.48	1.59
**PBIm14**	1.28	1.38	1.47	1.60

Table S2. Average lifetime (τ_{avg}) values of **PBIm4**, **PBIm10**, **PBIm12** and **PBIm14** in DMSO (λ_{ex} = 340 nm).

**PBIm*N* derivative** (50 μM)	**λ_{em}(nm)**	**τ_{avg} (ns)**
**PBIm4**	395	81.04
**PBIm10**	395	79.53
**PBIm12**	395	80.31
**PBIm14**	395	79.30

Table S3. Average lifetime (τ_{avg}) values of **PBIm4**, **PBIm10**, **PBIm12** and **PBIm14** in aqueous buffer (5 mM TRIS in 10 mM NaCl, pH 7.4, λ_{ex} = 340 nm).

**PBIm*N* derivative**	**λ_{em} (nm)**	**τ_{avg} (ns)**
**PBIm4** (50 μM)	395	15.08
**PBIm10** (100 μM)	395	31.9
	481	33.97
**PBIm12** (75 μM)	395	26.35
	485	34.44
**PBIm14** (50 μM)	395	15.83
	490	33.27

2. **Morphological study:**

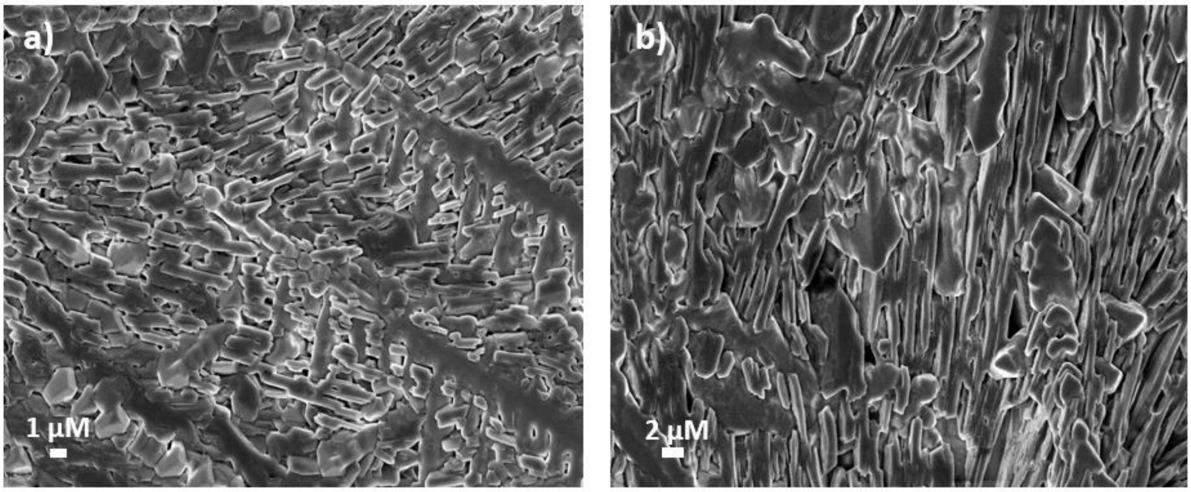

Figure S7. FESEM Images of (a) **PBIm10** and (b) **PBIm14**.

3. **Titration with phosphates in buffer:**

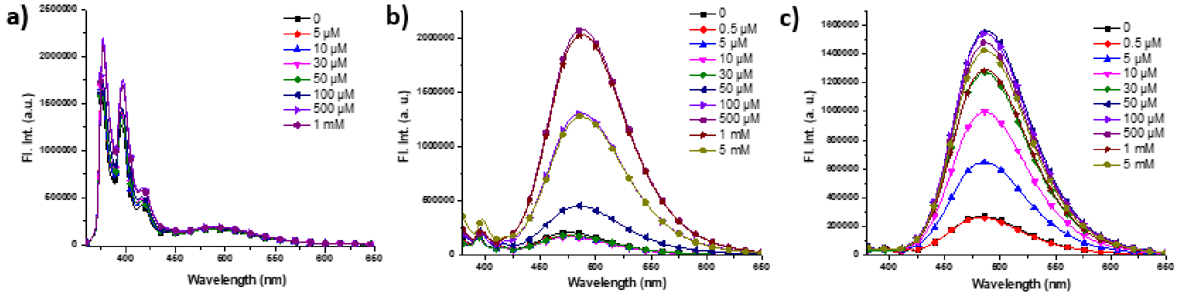

Figure S8. Emission spectral changes of (a) **PBIm4** (100 µM) (λ_{ex} = 350 nm), (b) **PBIm10** (100 µM) (λ_{ex} = 365 nm) and (c) **PBIm14** (30 µM) (λ_{ex} = 365 nm) upon addition of ATP in aqueous buffer (5 mM TRIS in 10 mM NaCl, pH 7.4).

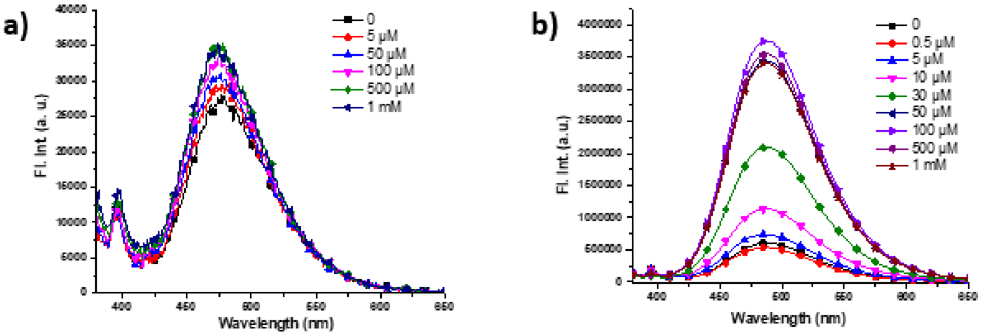

Figure S9. Emission spectral changes of **PBIm12** at (a) 1 µM and (b) 125 µM upon addition of ATP in aqueous buffer (5 mM TRIS in 10 mM NaCl, pH 7.4). (λ_{ex} = 365 nm).

Table S4. Average lifetime (τ_{avg}) values of **PBIm4**, **PBIm10**, **PBIm12** and **PBIm14** in presence of ATP in aqueous buffer (5 mM TRIS in 10 mM NaCl, pH 7.4). (λ_{ex} = 340 nm)

**PBIm*N* derivative**	**λ_{em} (nm)**	**τ_{avg} with ATP (100 µM) (ns)**
**PBIm4** (50 µM)	395	-
**PBIm10** (100 µM)	395	17.99
	481	35.81
**PBIm12** (75 µM)	395	18.73
	485	41.99
**PBIm14** (50 µM)	395	60.17
	490	40.96

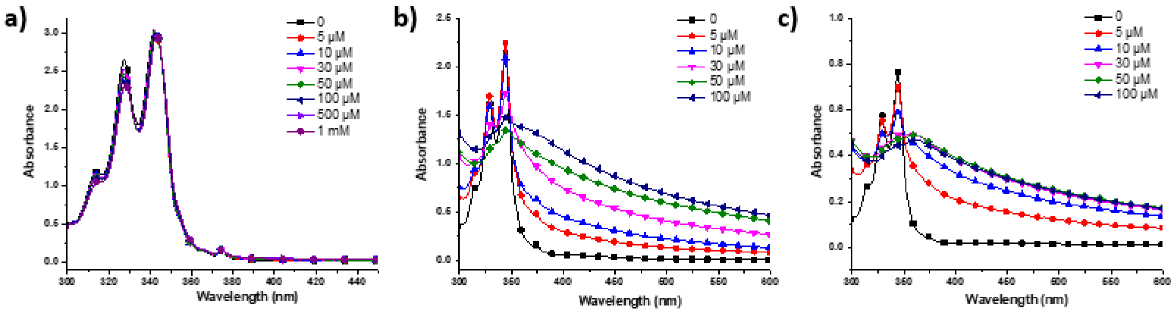

Figure S10. Absorption spectral changes of (a) **PBIm4** (100 µM), (b) **PBIm12** (75 µM) and (c) **PBIm14** (30 µM) upon addition of sodium salt of ATP in aqueous buffer (5 mM tris in 10 mM NaCl, pH 7.4).

Table S5. $A_{0\text{-}0}/A_{0\text{-}1}$ ratios for **PBIm12** (75 µM) and **PBIm14** (30 µM) in absence and in the presence of ATP in aqueous buffer (5 mM tris in 10 mM NaCl, pH 7.4)

**PBIm*N* derivatives (in buffer)**	**$A_{0\text{-}0}/A_{0\text{-}1}$ In absence of ATP**	**$A_{0\text{-}0}/A_{0\text{-}1}$ In presence of ATP (5 µM)**	**$A_{0\text{-}0}/A_{0\text{-}1}$ In presence of ATP (10 µM)**	**$A_{0\text{-}0}/A_{0\text{-}1}$ In presence of ATP (30 µM)**
**PBIm12** (75 µM)	1.318	1.326	1.317	1.217
**PBIm14** (30 µM)	1.325	1.269	1.188	1.103

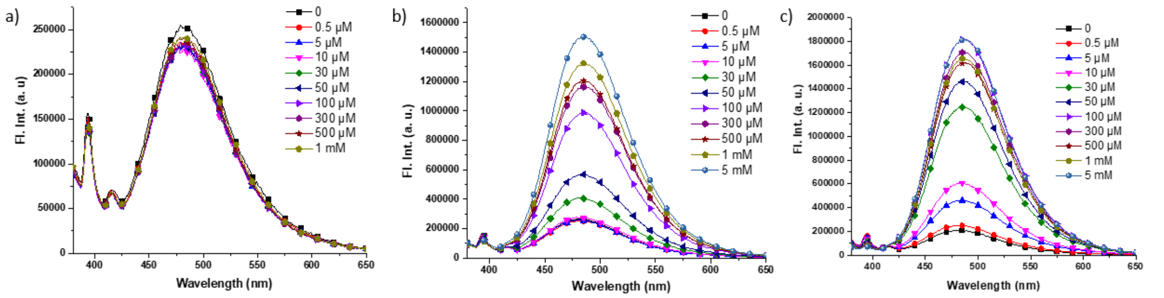

Figure S11. Emission spectral changes of **PBIm12** (75 μM) upon addition of (a) AMP (0.5 μM-1 mM), (b) ADP (0.5 μM-5 mM) and (c) PPi (0.5 μM-5 mM) in aqueous buffer (5 mM tris in 10 mM NaCl, pH 7.4) (λ_{ex} = 365 nm).

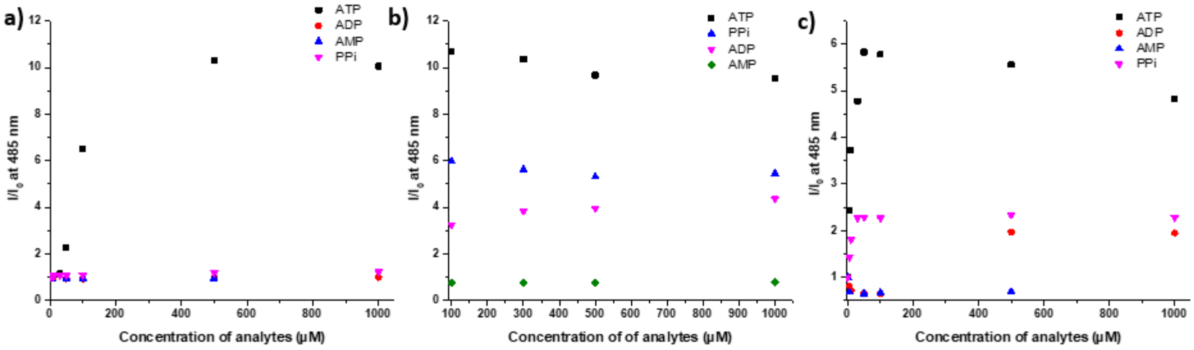

Figure S12. Change in I/I0 of (a) **PBIm10** (100 μM), (b) **PBIm12** (75 μM) and (b) **PBIm14** (30 μM) upon addition of different analytes in aqueous buffer (5 mM tris in 10 mM NaCl, pH 7.4) (λ_{ex} = 365 nm).

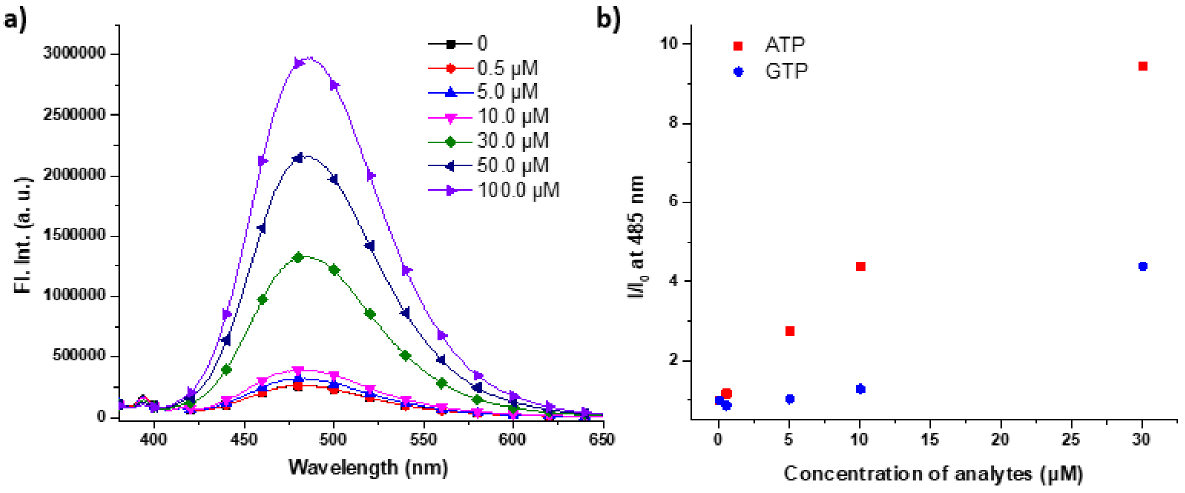

Figure S13. (a) Emission spectral changes of **PBIm12** (75 μM) upon addition of GTP (0.5 μM-100 μM) in aqueous buffer (5 mM tris in 10 mM NaCl, pH 7.4) (λ_{ex} = 365 nm). (b) Change in I/I0 of **PBIm12** (75 μM) upon addition of ATP and GTP in aqueous buffer.

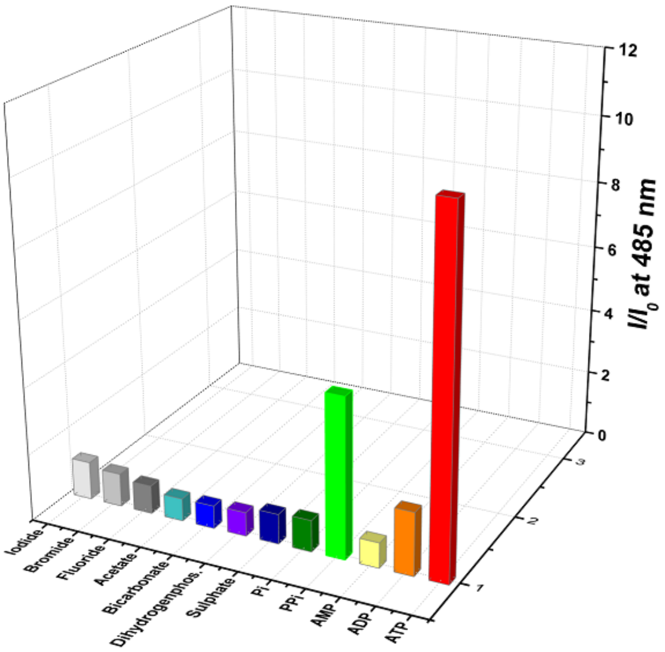

Figure S14. Bar diagram of **PBIm12** (75 µM) upon addition of different anions (50 µM) in aqueous buffer (5 mM tris in 10 mM NaCl, pH 7.4) (λ_{ex} = 365 nm).

4. Lifetime measurements:

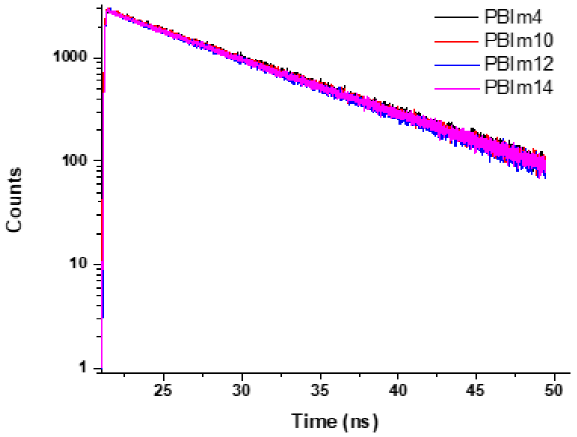

Figure S15. Time resolved decay curves of **PBIm4**, **PBIm10**, **PBIm12** and **PBIm14** (50 µM of each) in DMSO (λ_{ex} = 340 nm).

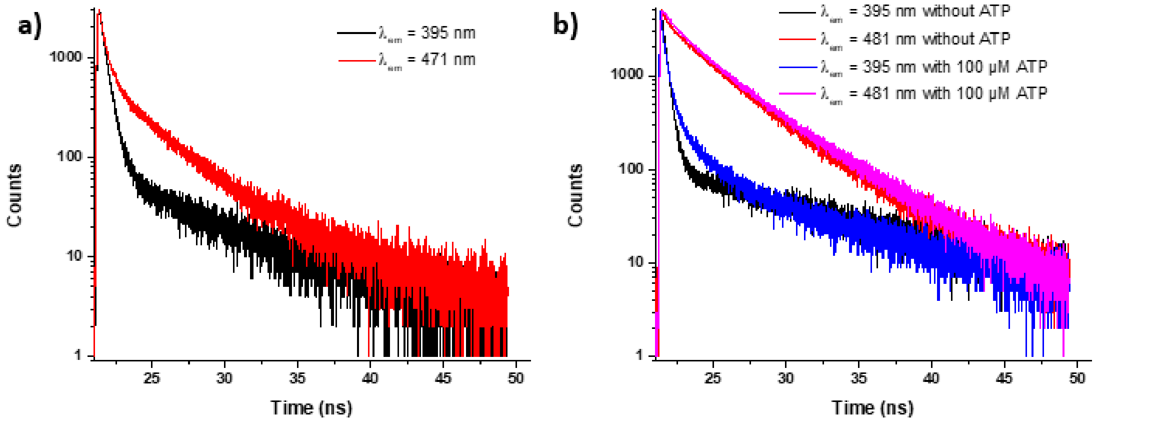

Figure S16. (a) Time resolved decay curves of **PBIm4** (100 µM) in aqueous buffer. (b) Time resolved decay curves of **PBIm10** (100 µM) in absence and in presence of ATP in aqueous buffer. (λ_{ex} = 340 nm).

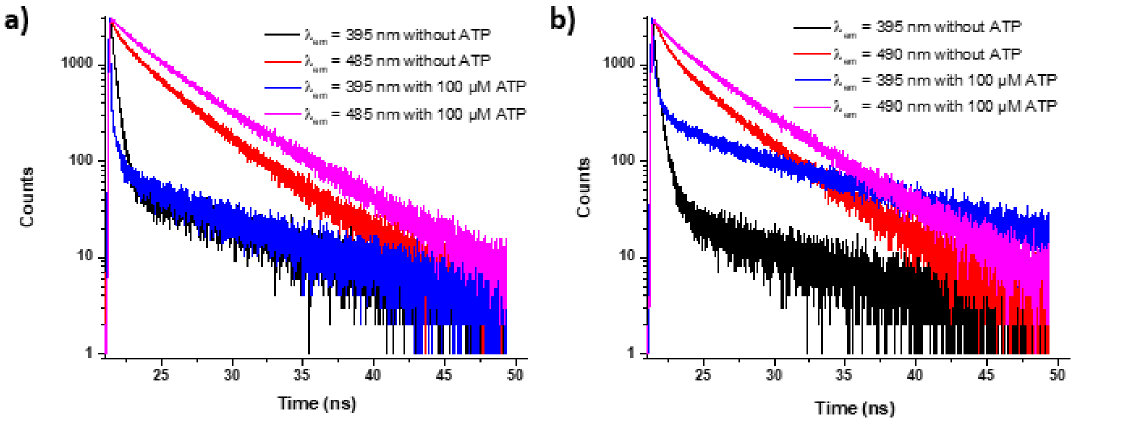

Figure S17. Time resolved decay curves of (a) **PBIm12** (75 µM) and (b) **PBIm14** (30 µM) in absence and in presence of ATP in aqueous buffer. (λ_{ex} = 340 nm)

5. Size and zeta potential measurements:

A. Size of PBIm10 (150 µM)

Results

			Size (d.nm):	% Intensity:	St Dev (d.n...
Z-Average (d.nm):	602.6	Peak 1:	545.0	98.6	145.7
PdI:	0.355	Peak 2:	5560	1.4	6.104e-5
Intercept:	0.937	Peak 3:	0.000	0.0	0.000

Result quality : Refer to quality report

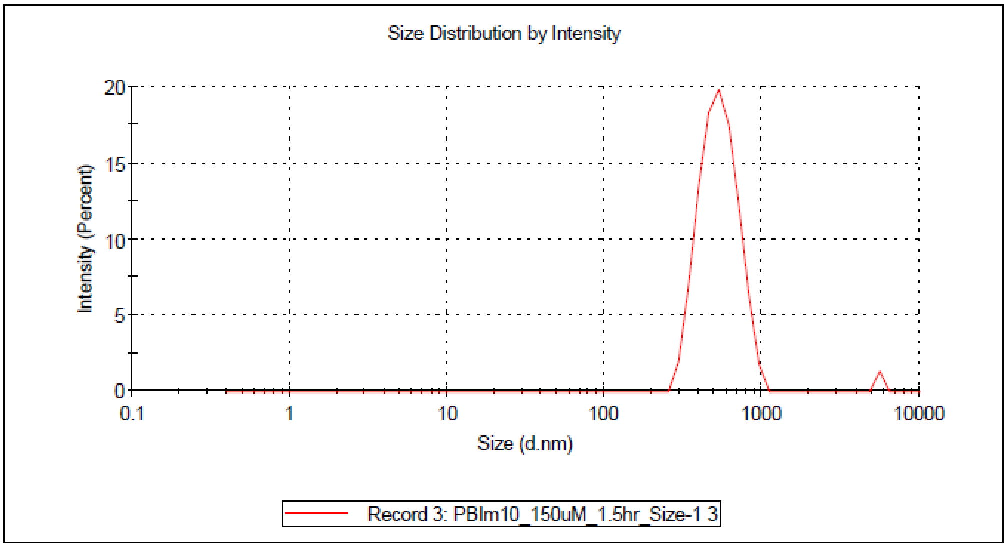

B. Size of PBIm12 (75 µM)

Results

			Size (d.nm):	% Intensity:	St Dev (d.n...
Z-Average (d.nm):	689.2	Peak 1:	818.5	85.3	432.9
PdI:	0.450	Peak 2:	4854	8.4	742.0
Intercept:	0.956	Peak 3:	153.7	6.3	39.09

Result quality : Good

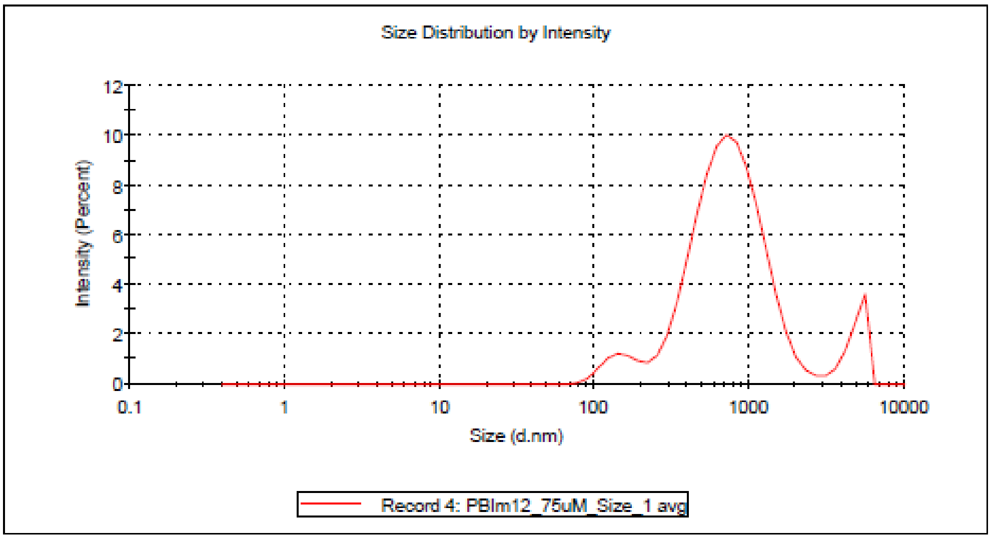

C. Size of PBIm14 (50 μM)

Results

			Size (d.nm):	% Intensity:	St Dev (d.n...
Z-Average (d.nm):	621.3	Peak 1:	118.8	94.1	20.16
PdI:	0.657	Peak 2:	5.263	5.9	1.157
Intercept:	0.566	Peak 3:	0.000	0.0	0.000

Result quality : Refer to quality report

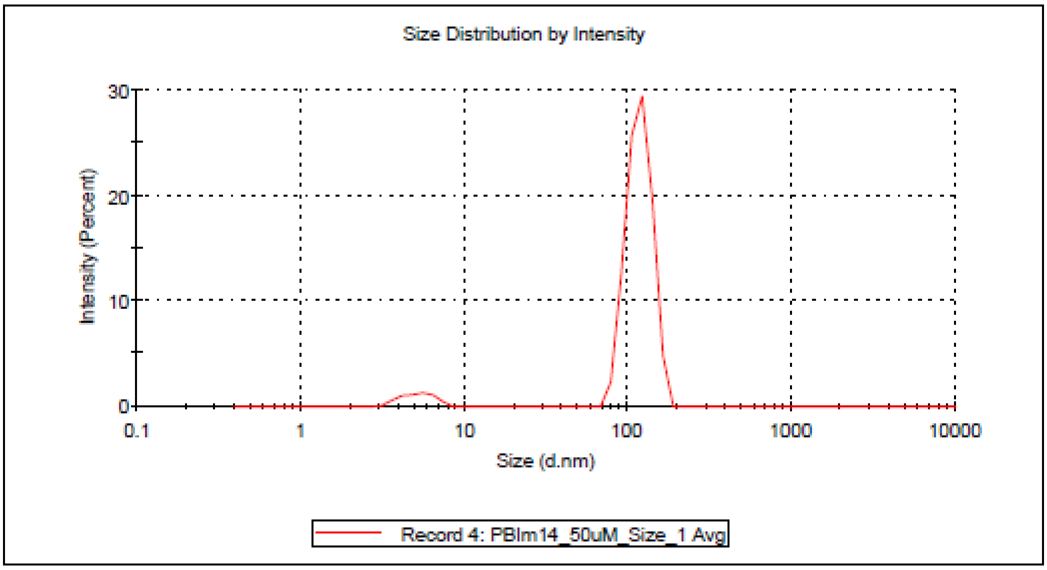

D. Size of PBIm10 (150 μM) with ATP (100 μM)

Results

			Size (d.nm):	% Intensity:	St Dev (d.n...
Z-Average (d.nm):	1582	Peak 1:	673.1	100.0	89.79
PdI:	0.796	Peak 2:	0.000	0.0	0.000
Intercept:	0.944	Peak 3:	0.000	0.0	0.000

Result quality : Refer to quality report

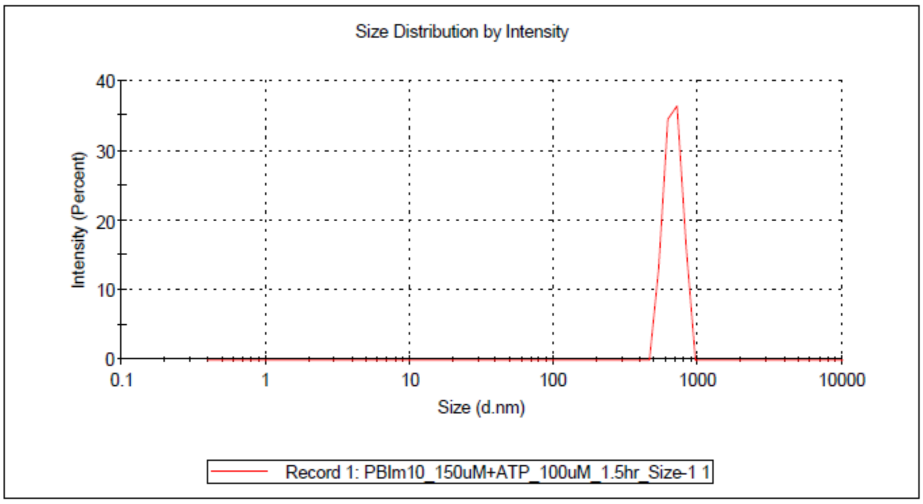

E. Size of PBIm12 (75 µM) with ATP (50 µM)

Results

			Size (d.nm):	% Intensity:	St Dev (d.n...
Z-Average (d.nm):	1008	Peak 1:	812.3	91.8	183.6
PdI:	0.607	Peak 2:	158.8	8.2	34.93
Intercept:	0.934	Peak 3:	0.000	0.0	0.000

Result quality : Refer to quality report

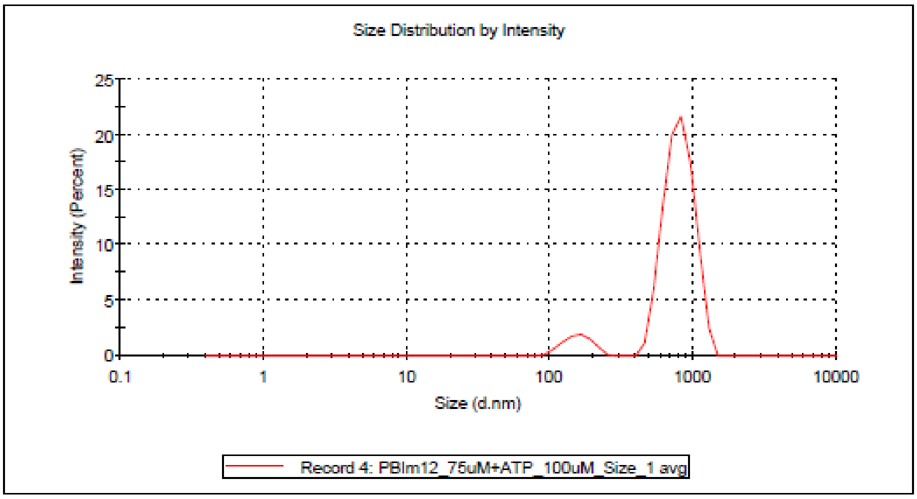

F. Size of PBIm14 (50 µM) with ATP (50 µM)

Results

			Size (d.nm):	% Intensity:	St Dev (d.n...
Z-Average (d.nm):	561.1	Peak 1:	616.2	98.9	228.1
PdI:	0.184	Peak 2:	4973	1.1	621.2
Intercept:	0.936	Peak 3:	0.000	0.0	0.000

Result quality : Good

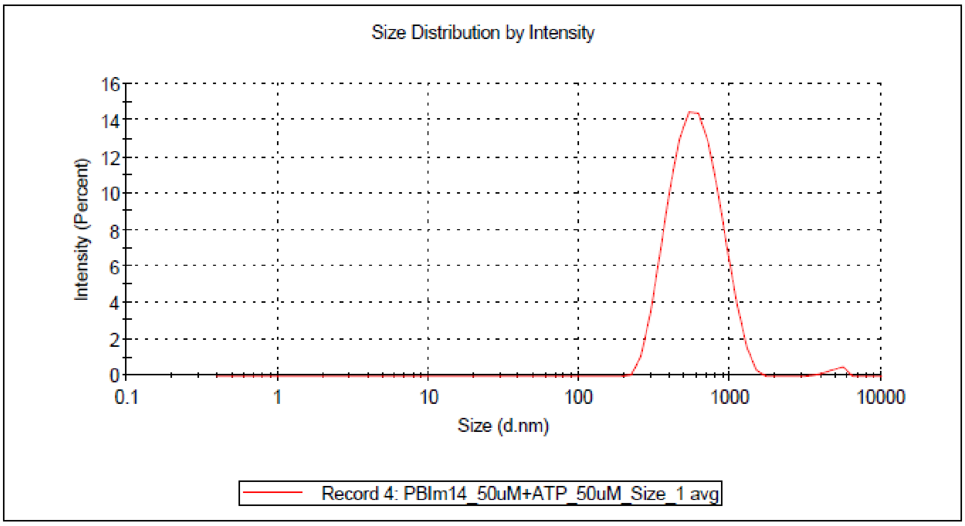

G. Zeta of PBIm10 (150 µM)

Results

			Mean (mV)	Area (%)	St Dev (mV)
Zeta Potential (mV):	2.74	Peak 1:	2.74	100.0	3.88
Zeta Deviation (mV):	3.88	Peak 2:	0.00	0.0	0.00
Conductivity (mS/cm):	1.25	Peak 3:	0.00	0.0	0.00
Result quality :	Good				

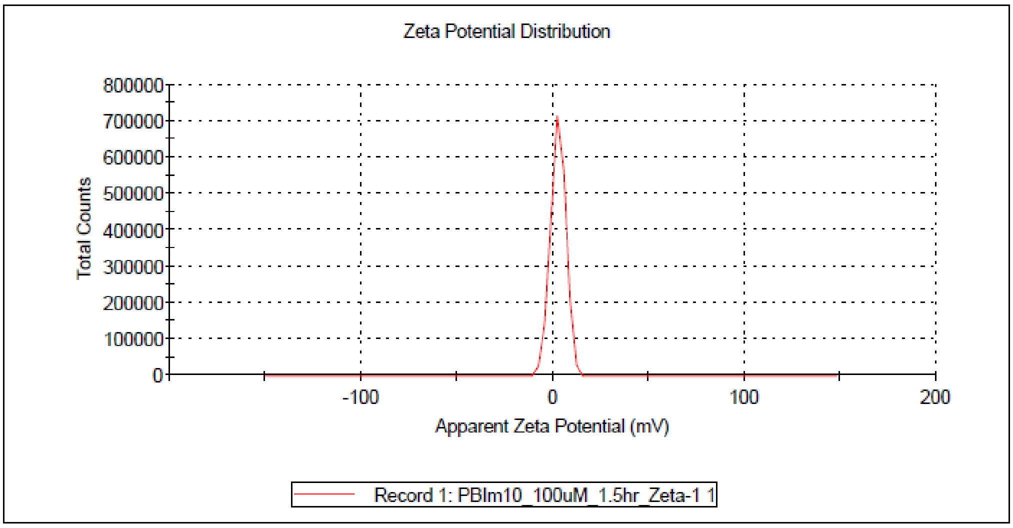

H. Zeta of PBIm12 (75 µM)

Results

			Mean (mV)	Area (%)	St Dev (mV)
Zeta Potential (mV):	2.41	Peak 1:	2.24	99.8	5.64
Zeta Deviation (mV):	5.53	Peak 2:	33.4	0.2	0.00
Conductivity (mS/cm):	1.39	Peak 3:	0.00	0.0	0.00
Result quality :	Good				

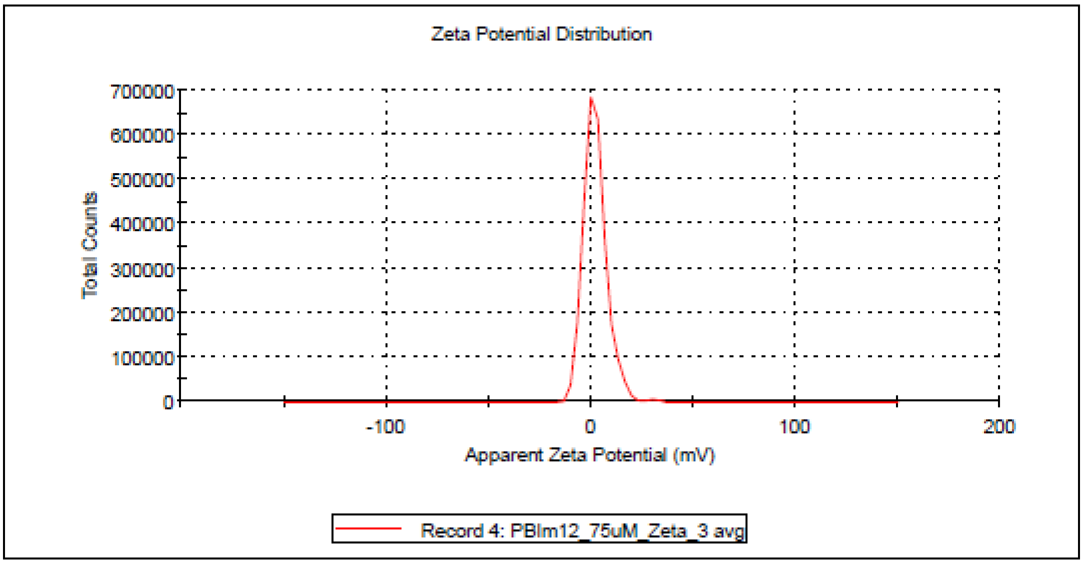

I. Zeta of PBIm14 (50 μM)

Results

			Mean (mV)	Area (%)	St Dev (mV)
**Zeta Potential (mV):**	3.35	**Peak 1:**	2.91	100.0	6.13
**Zeta Deviation (mV):**	6.06	**Peak 2:**	0.00	0.0	0.00
**Conductivity (mS/cm):**	1.39	**Peak 3:**	0.00	0.0	0.00
**Result quality :**	Good				

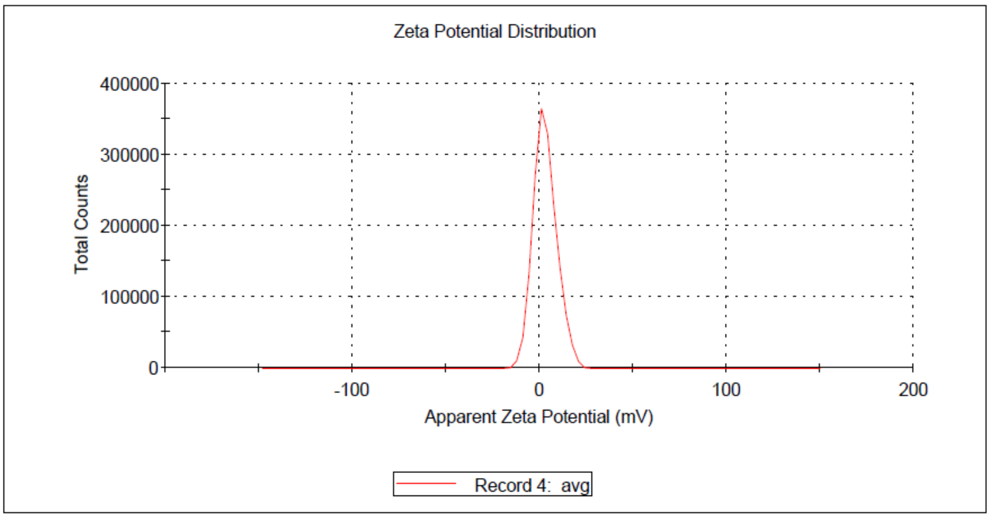

J. Zeta of PBIm10 (150 μM) with ATP (100 μM)

Results

			Mean (mV)	Area (%)	St Dev (mV)
**Zeta Potential (mV):**	-10.1	**Peak 1:**	-10.1	100.0	4.64
**Zeta Deviation (mV):**	4.64	**Peak 2:**	0.00	0.0	0.00
**Conductivity (mS/cm):**	1.25	**Peak 3:**	0.00	0.0	0.00
**Result quality :**	Good				

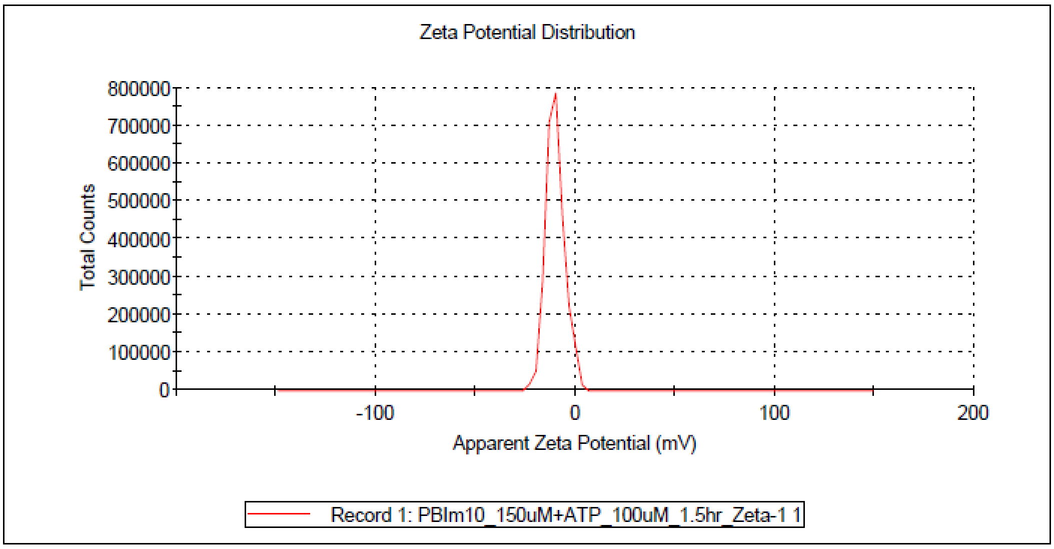

K. Zeta of PBIm12 (75 µM) with ATP (50 µM)

Results

			Mean (mV)	Area (%)	St Dev (mV)
Zeta Potential (mV):	-8.02	Peak 1:	-7.98	100.0	4.88
Zeta Deviation (mV):	4.61	Peak 2:	0.00	0.0	0.00
Conductivity (mS/cm):	1.39	Peak 3:	0.00	0.0	0.00
Result quality :	Good				

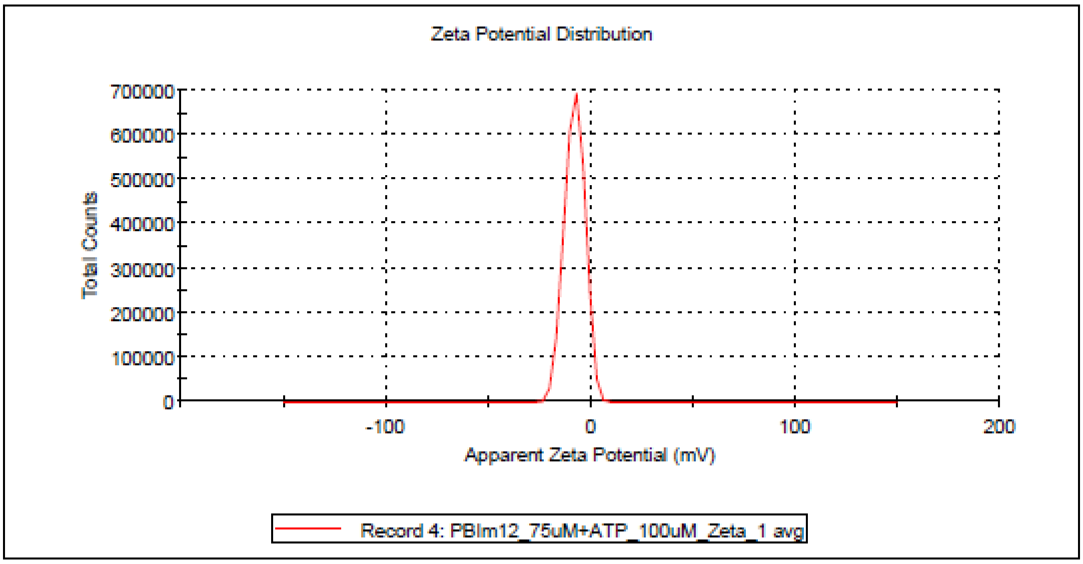

L. Zeta of PBIm14 (50 µM) with ATP (50 µM)

Results

			Mean (mV)	Area (%)	St Dev (mV)
Zeta Potential (mV):	-4.98	Peak 1:	-4.99	100.0	6.84
Zeta Deviation (mV):	6.67	Peak 2:	0.00	0.0	0.00
Conductivity (mS/cm):	1.44	Peak 3:	0.00	0.0	0.00
Result quality :	Good				

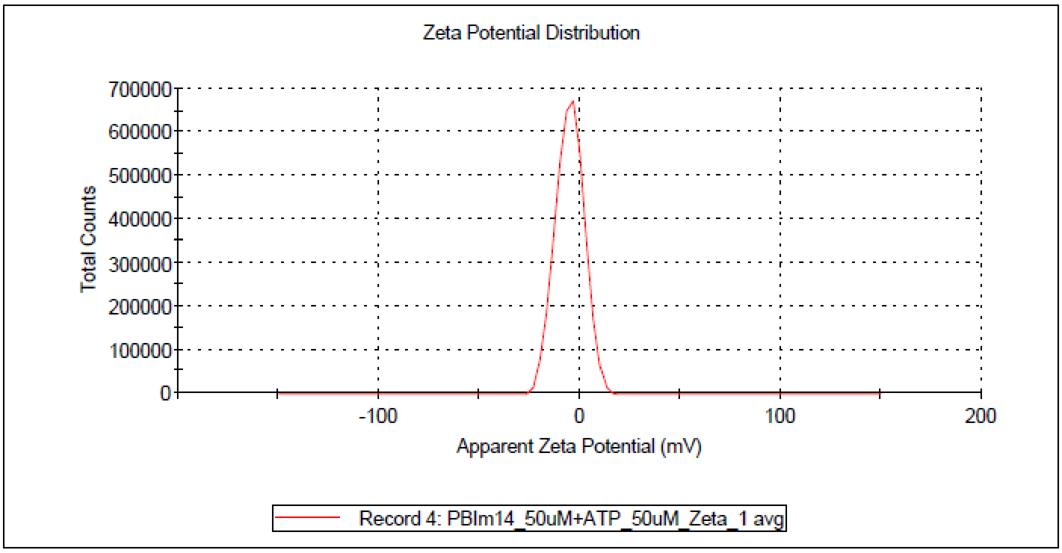

6. NMR spectra:

A. ^{1}H NMR spectra of **1** (400 MHz, $CDCl_3$):

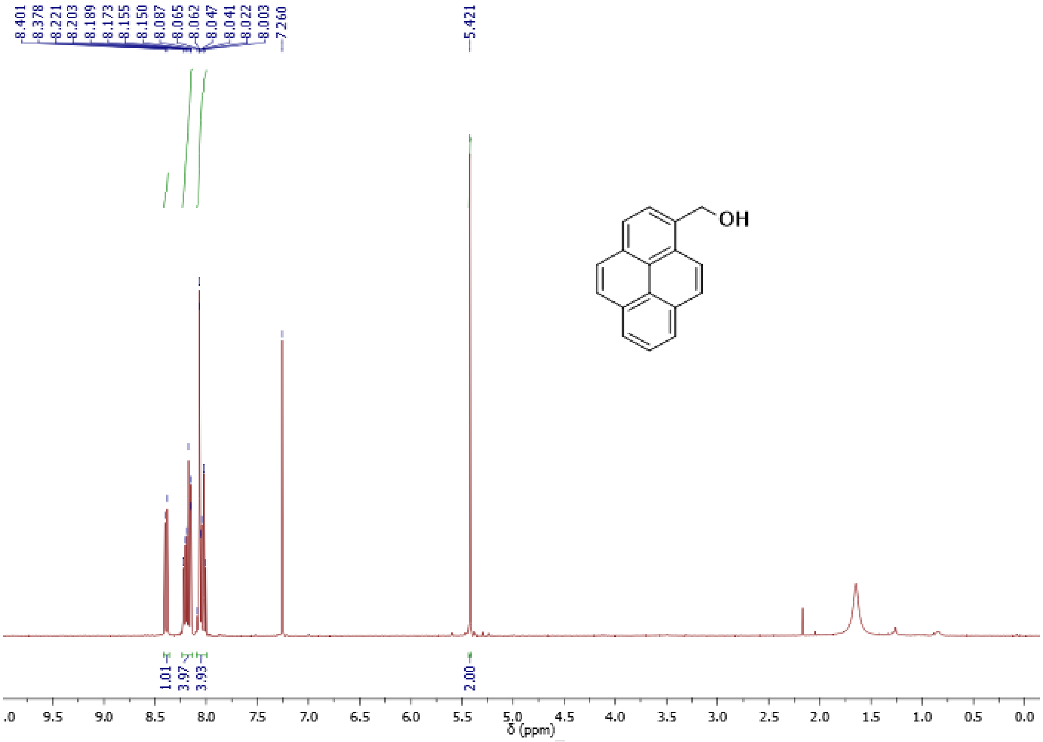

B. ^{1}H NMR spectra of **2** (400 MHz, $CDCl_3$):

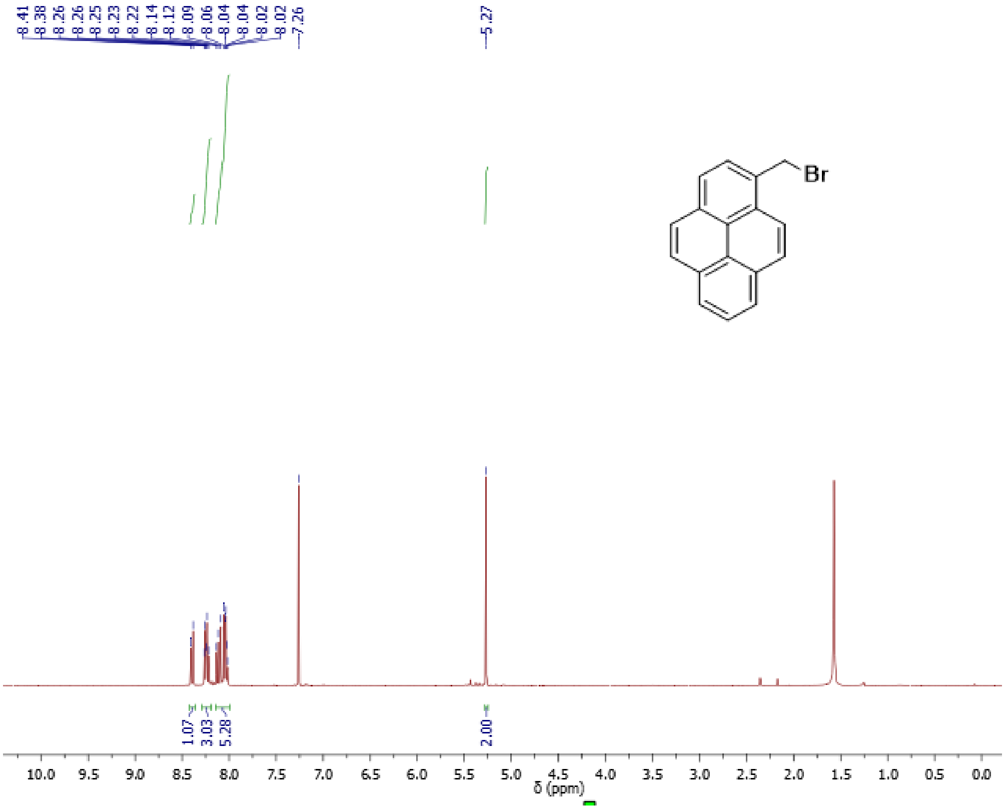

C. ^{1}H NMR spectra of **Di(1H-imidazol-1-yl)methane** (400 MHz, DMSO-D$_6$)

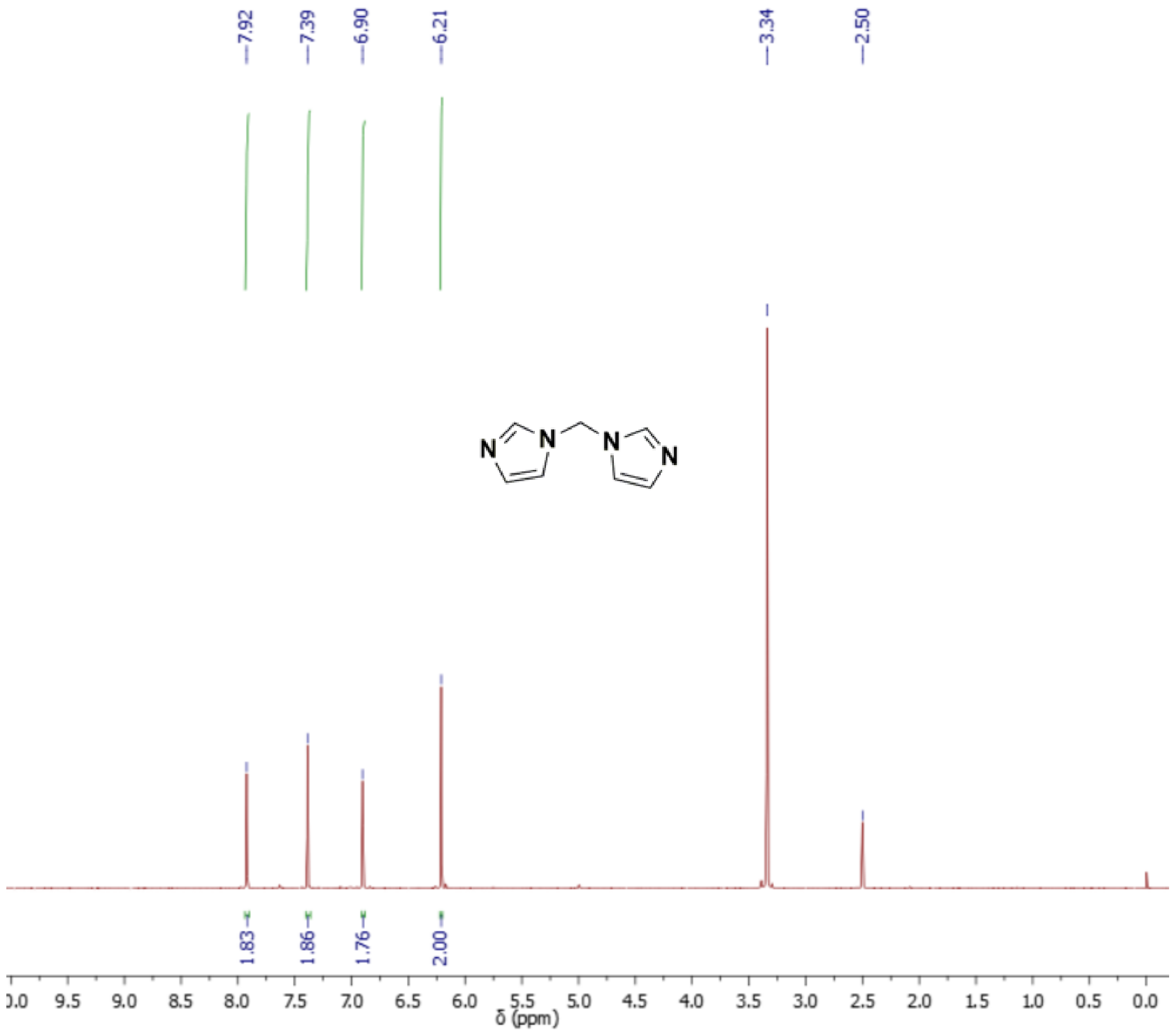

D. ^{1}H NMR spectra of **BIm4** (400 MHz, DMSO-D_6)

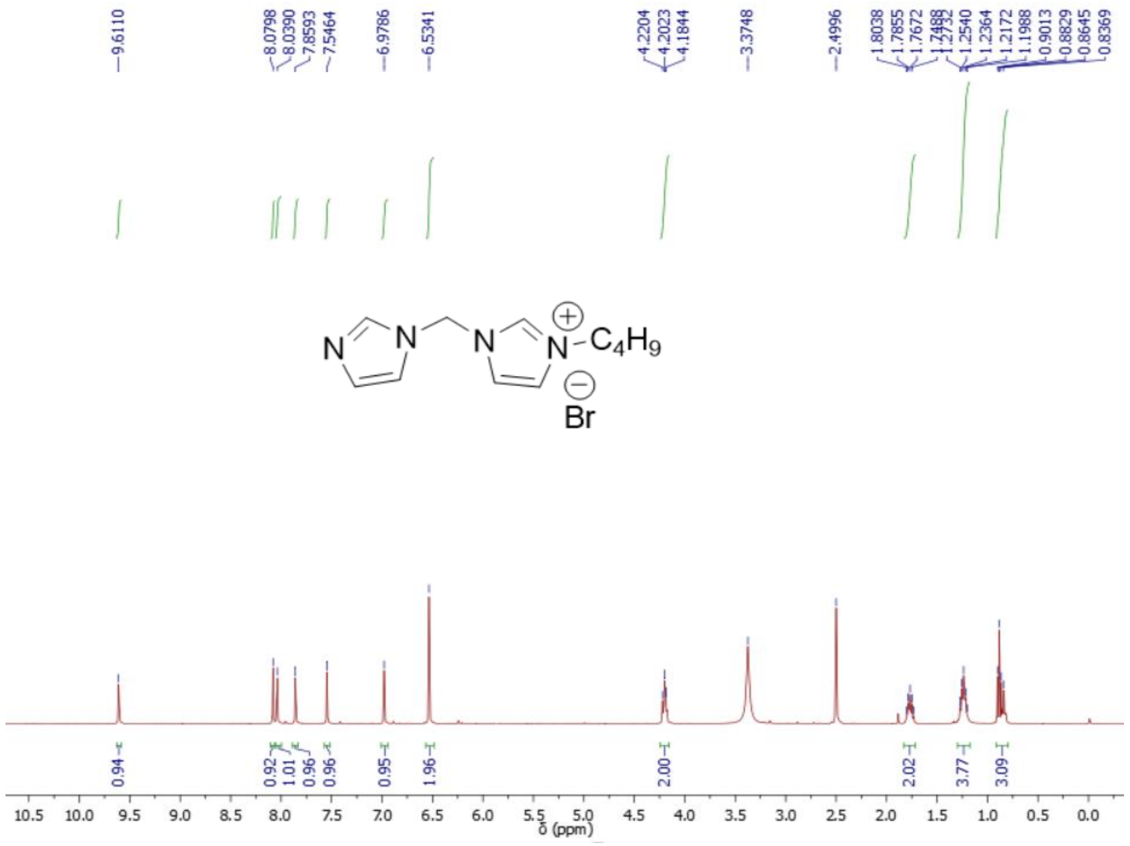

E. ^{13}C NMR spectra of **BIm4** (100 MHz, DMSO-D_6)

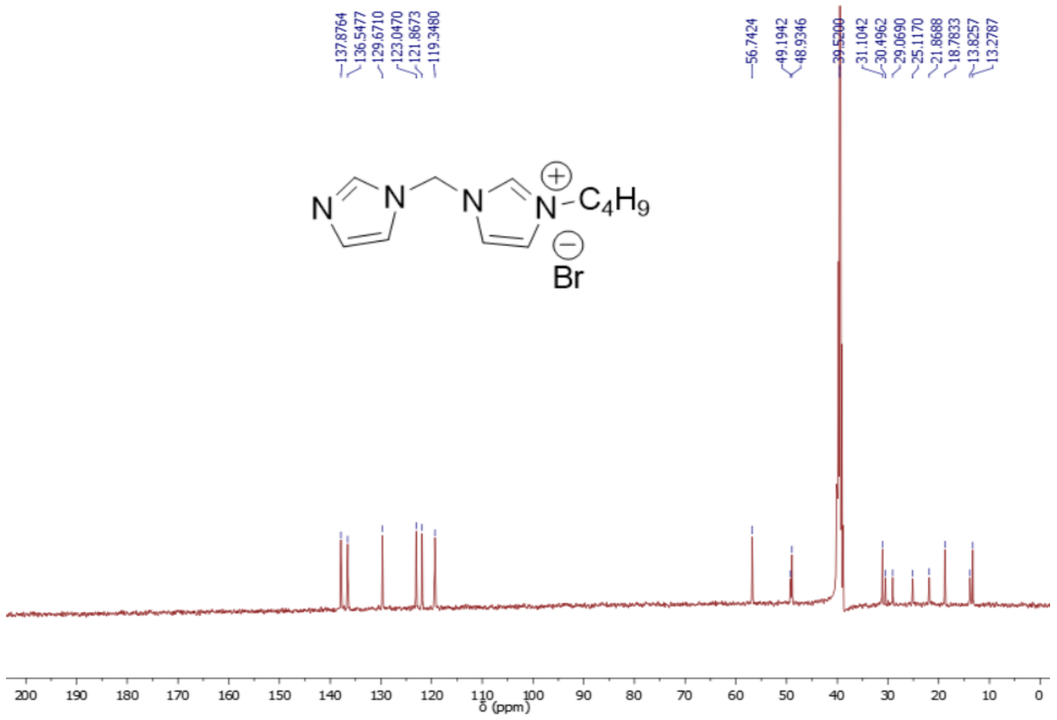

F. ^{1}H NMR spectra of **BIm10** (400 MHz, $CDCl_3$)

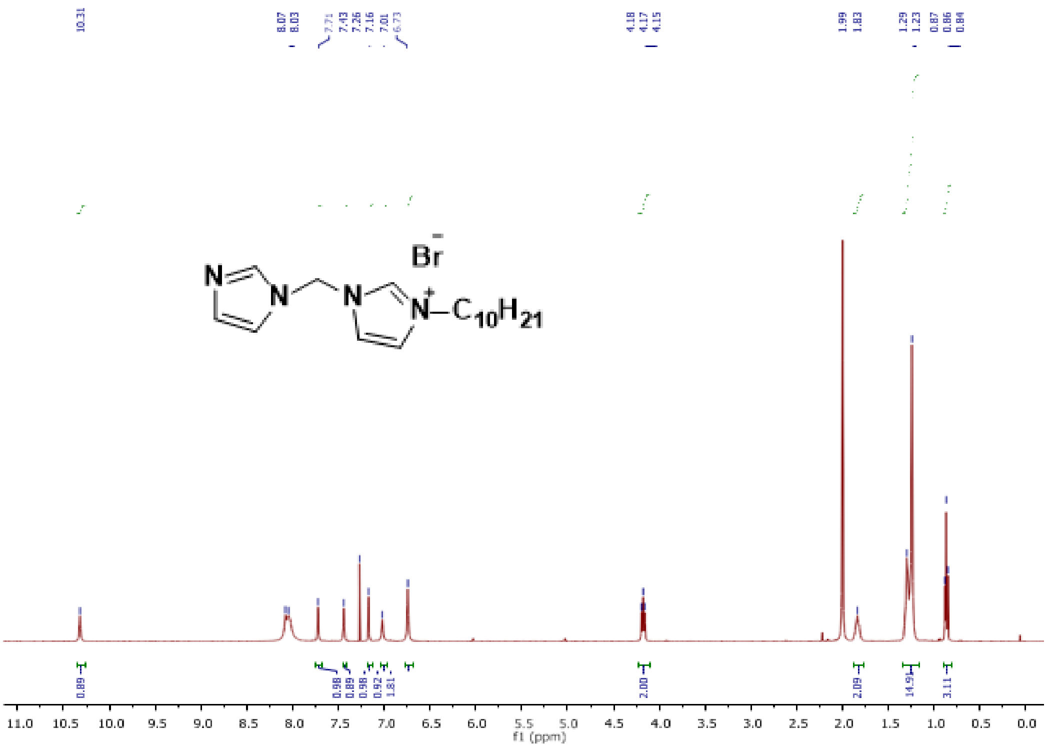

G. ^{13}C NMR spectra of **BIm10** (100 MHz, $CDCl_3$)

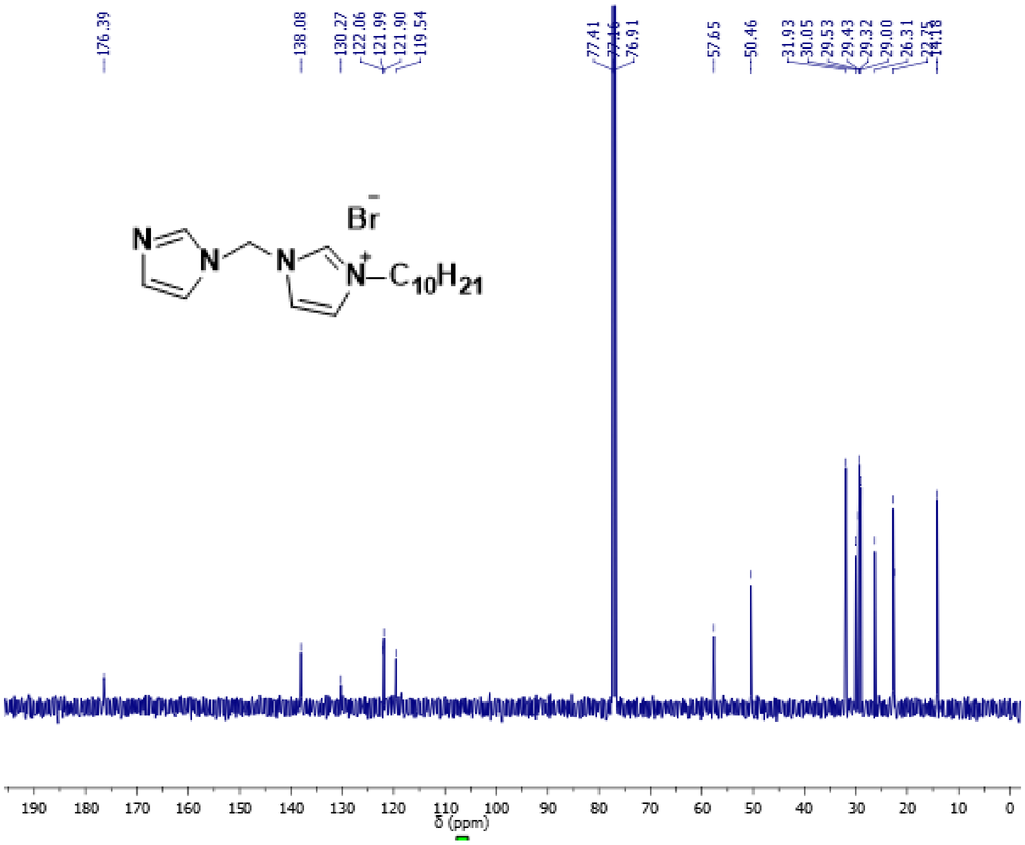

H. ^{1}H NMR spectra of **BIm12** (400 MHz, DMSO-D$_6$)

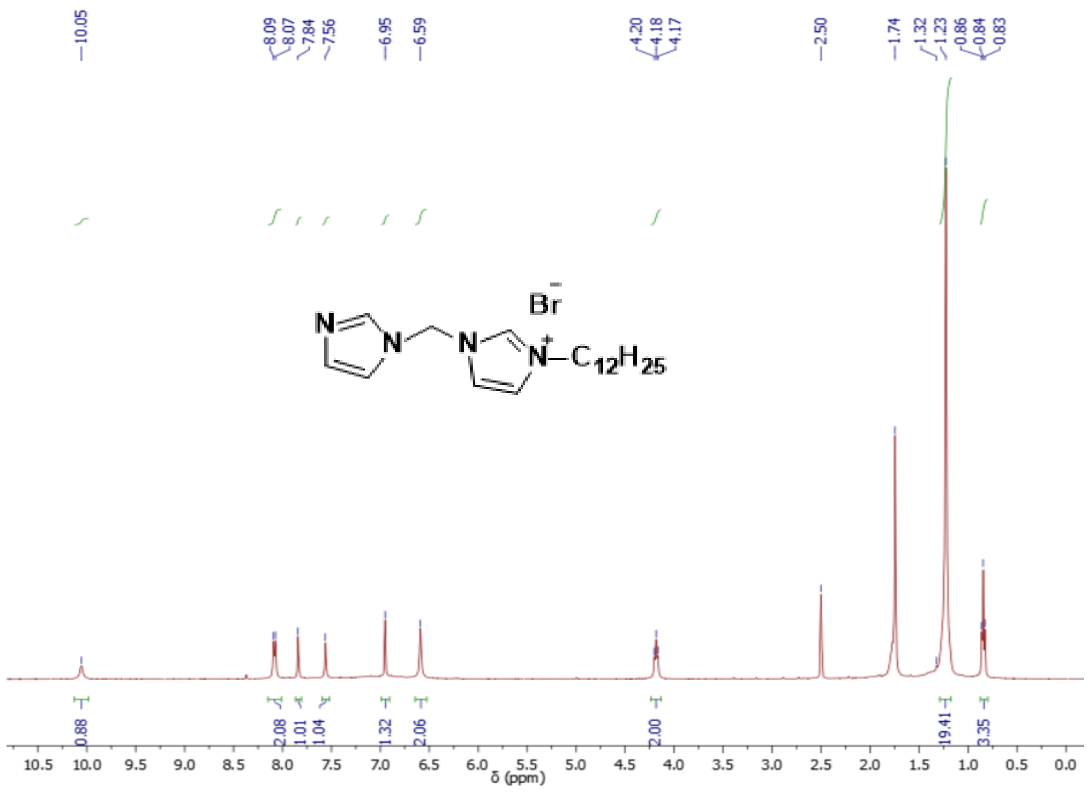

I. ^{13}C NMR spectra of **BIm12** (100 MHz, DMSO-D$_6$)

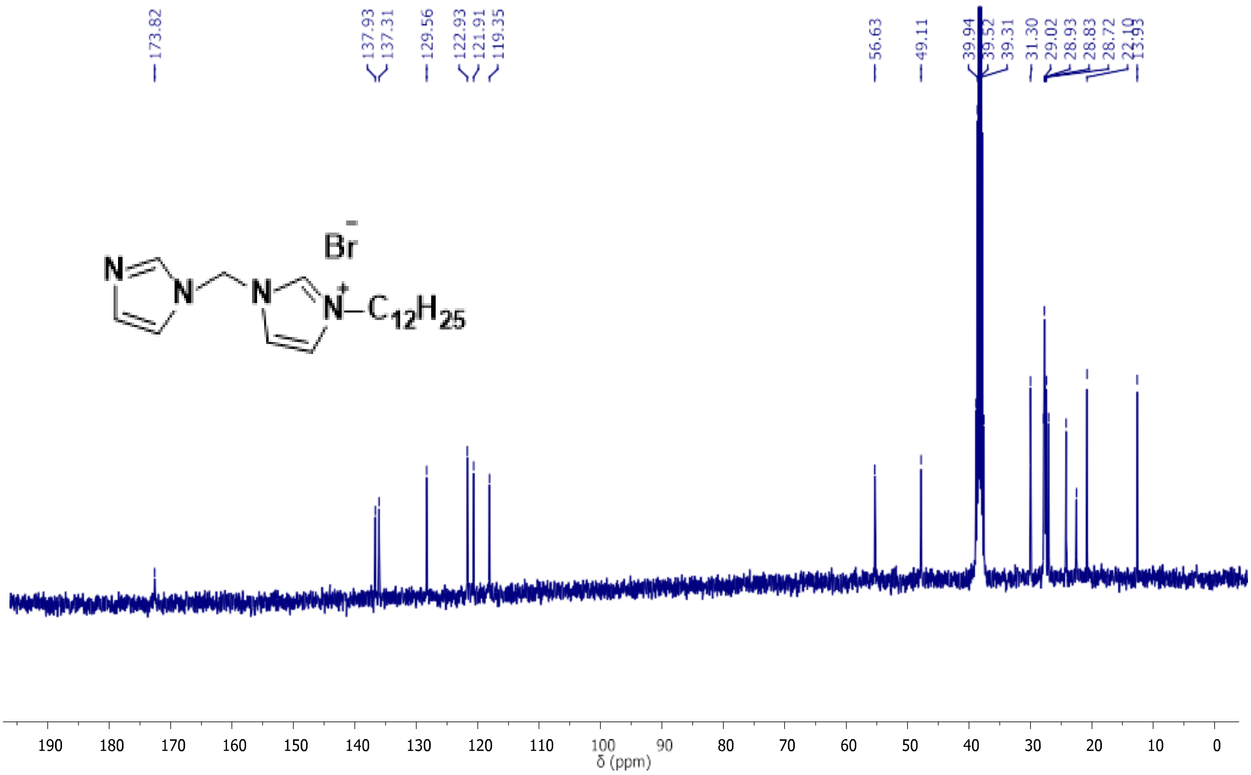

J. ^{1}H NMR spectra of **BIm14** (400 MHz, $CDCl_3$)

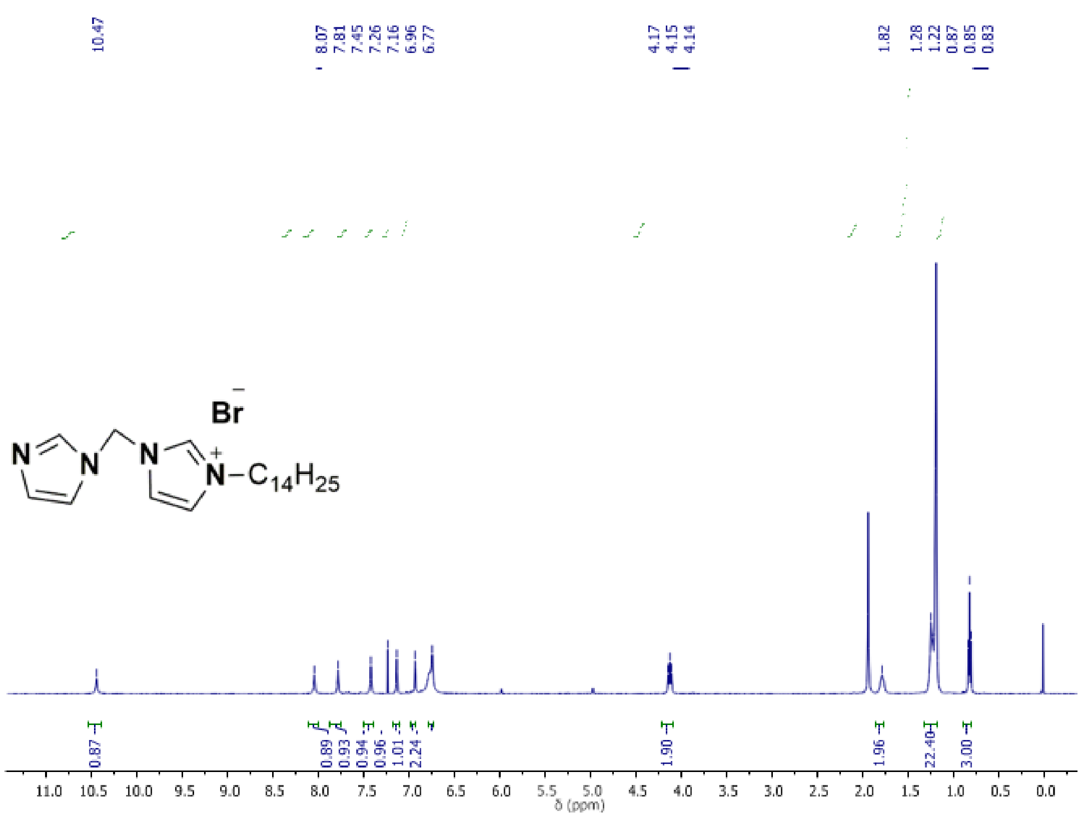

K. ^{13}C NMR spectra of **BIm14** (100 MHz, DMSO-D_6)

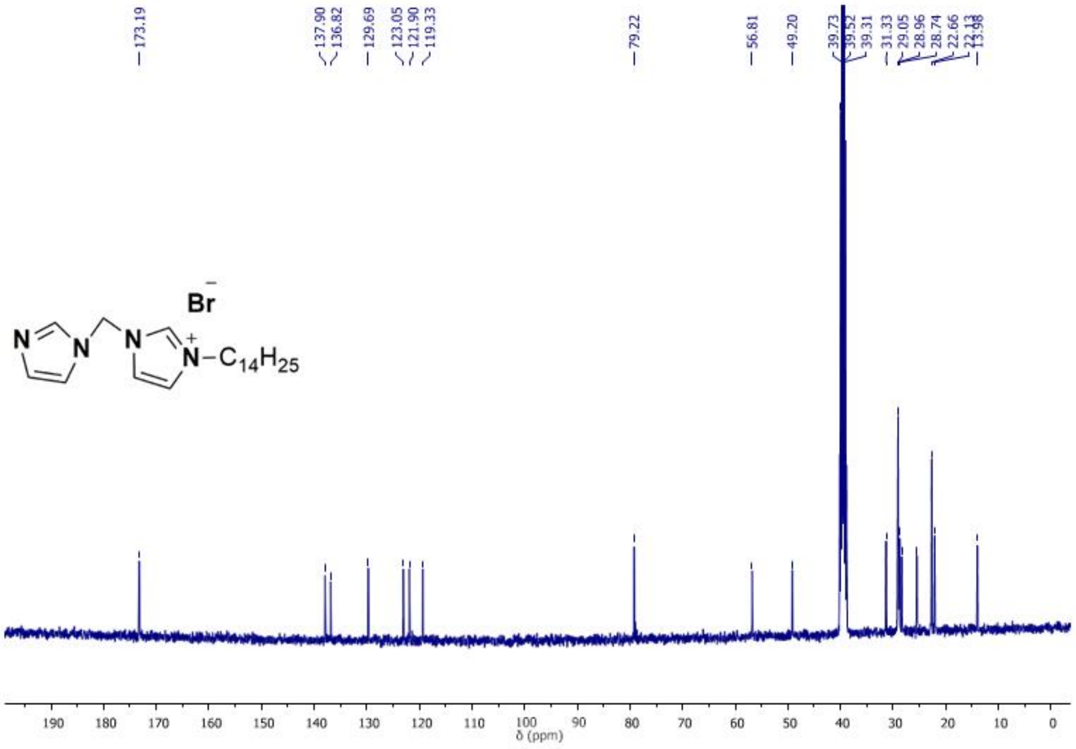

L. ^{1}H NMR spectra of **PBIm4** (400 MHz, DMSO-D$_6$)

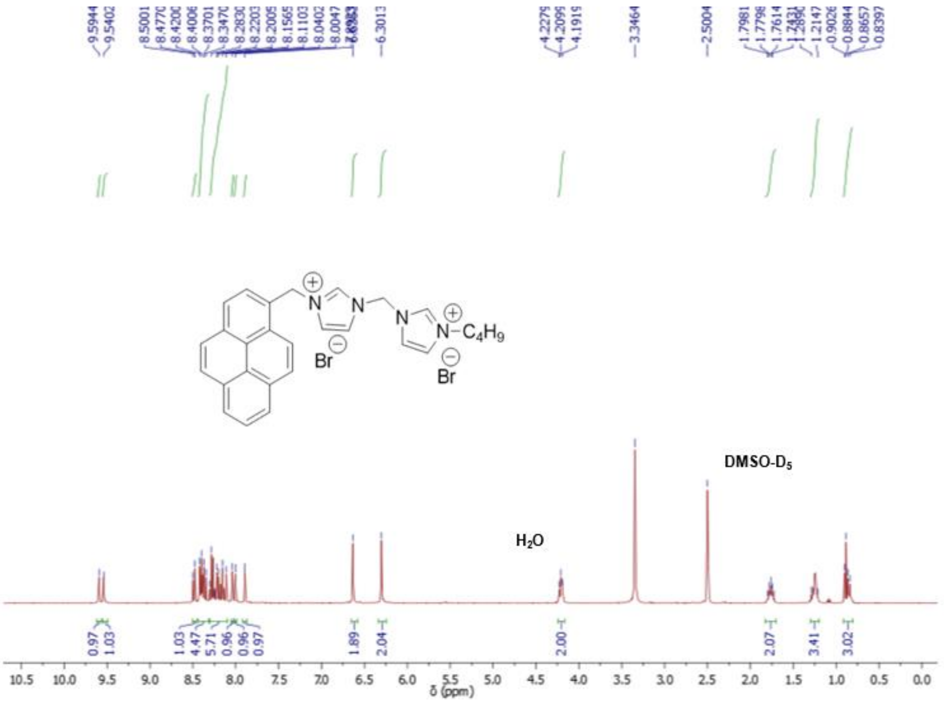

M. ^{13}C NMR spectra of **PBIm4** (100 MHz, DMSO-D$_6$)

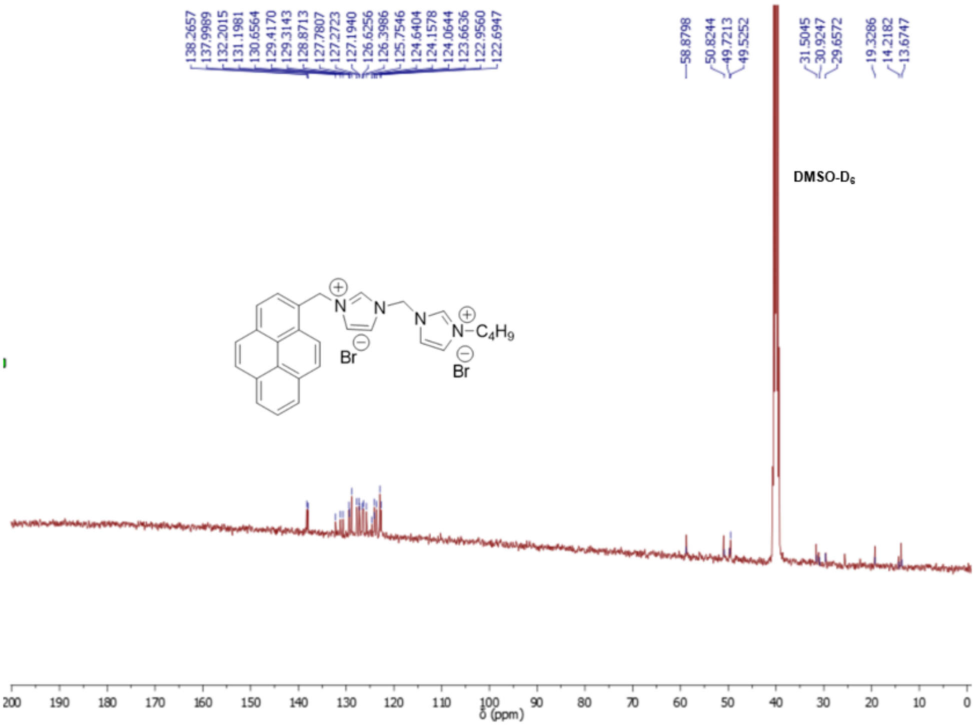

N. ^{1}H NMR spectra of **PBIm10** (400 MHz, DMSO-D_6)

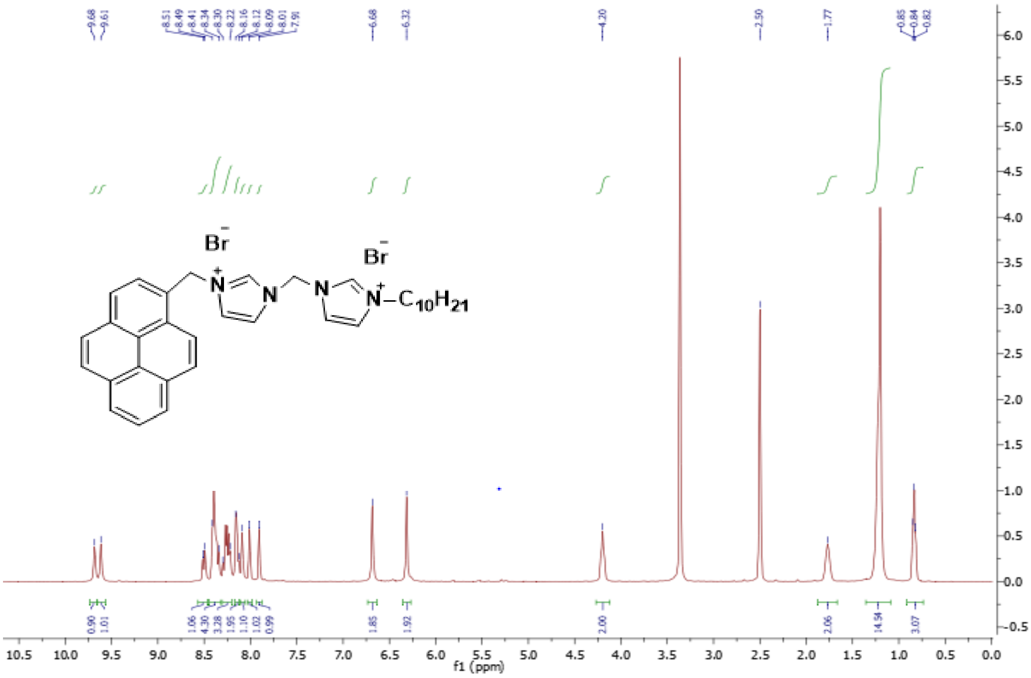

O. ^{13}C NMR spectra of **PBIm10** (100 MHz, DMSO-D_6)

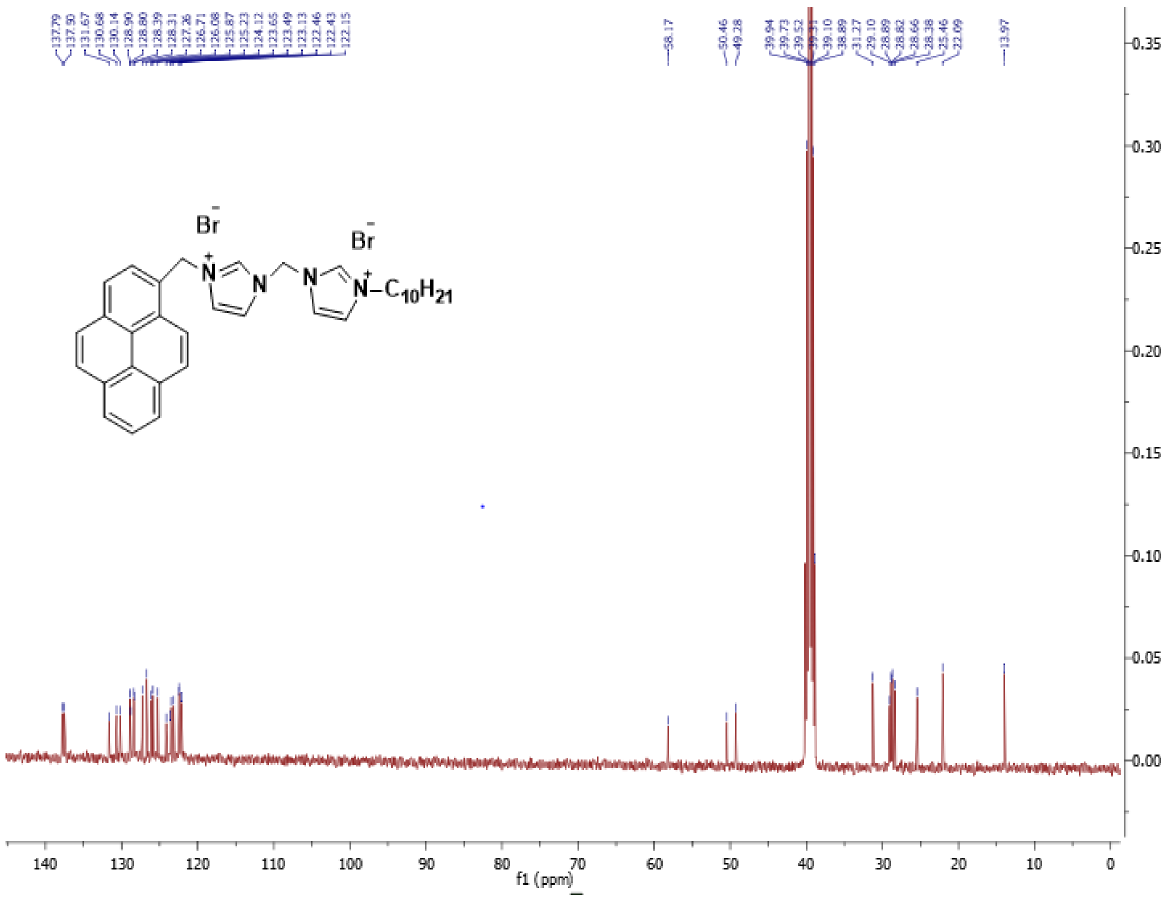

P. ^{1}H NMR spectra of **PBIm12** (400 MHz, DMSO-D$_6$)

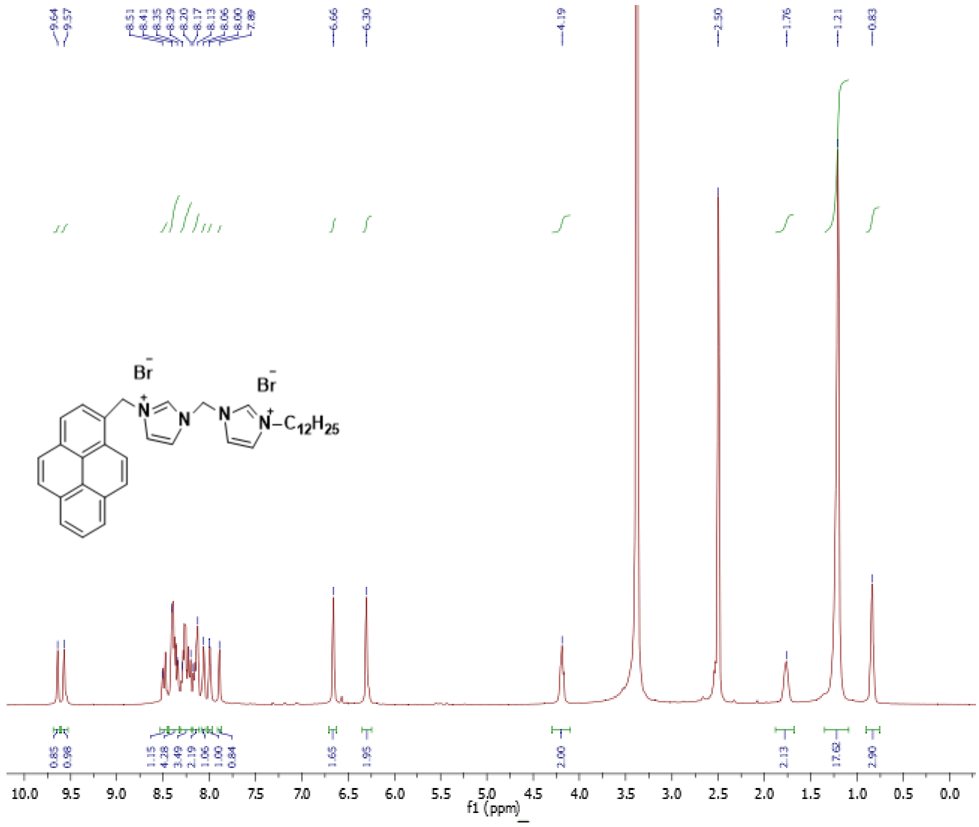

Q. ^{13}C NMR spectra of **PBIm12** (100 MHz, DMSO-D$_6$)

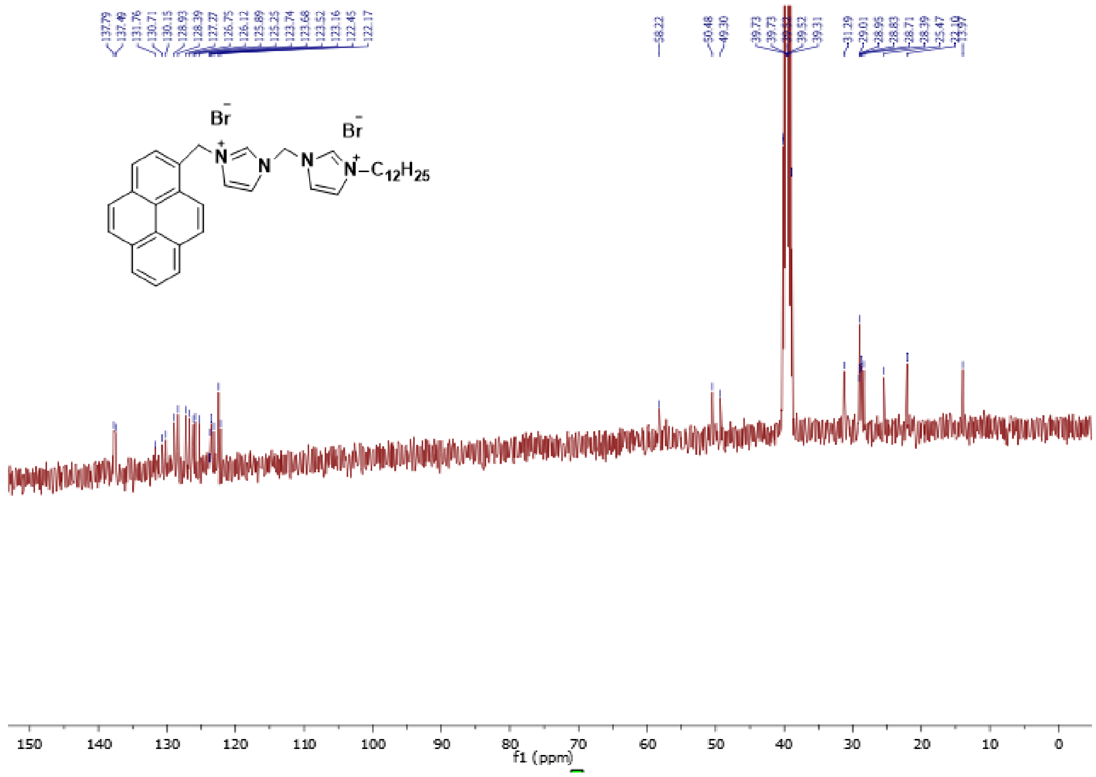

R. ^{1}H NMR spectra of **PBIm14** (400 MHz, DMSO-D_6)

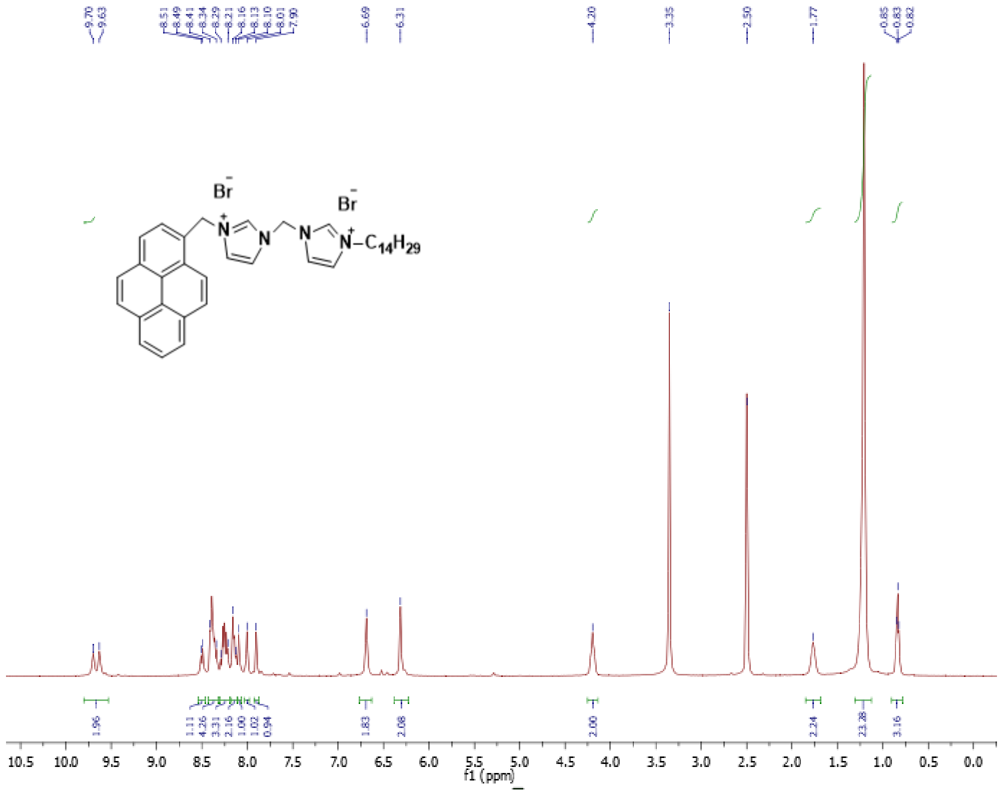

S. ^{13}C NMR spectra of **PBIm14** (100 MHz, DMSO-D_6)

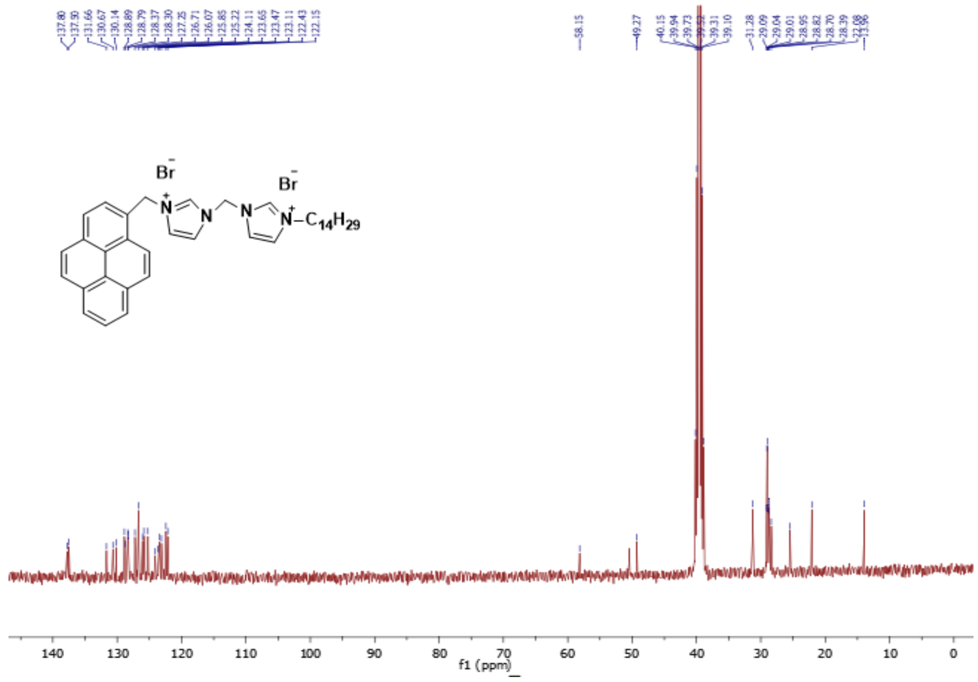

Supplement: File 1 — Synthetic procedures, additional spectroscopic, microscopic and dynamic light scattering data (figures and tables) and NMR characterization. [file Beilstein_J_Org_Chem-16-2728-s001.pdf]
